# Kinetic study on the reaction of sodium nitrite with neurotransmitters secreted in the stomach

**DOI:** 10.1038/s41598-023-42759-x

**Published:** 2023-09-21

**Authors:** Mario González-Jiménez, M. Pilar García-Santos, Blanca Bermejo Tesón, Ángel L. Fuentes de Arriba, Jorge Arenas Valgañón, Emilio Calle, Julio Casado

**Affiliations:** 1 Departamento de Química Física, Universidad de Salamanca, Plaza de los Caídos, 1-5, 37008 Salamanca, Spain; 2 Departamento de Química Orgánica, Universidad de Salamanca, Plaza de los Caídos, 1-5, 37008 Salamanca, Spain; 3 Present Address: School of Chemistry, University of Glasgow, Glasgow, G12 8QQ UK

**Keywords:** Reaction kinetics and dynamics, Chemical biology

## Abstract

Nitroso-compounds are potentially mutagenic and carcinogenic compounds due to their ability to alkylate DNA bases. One of the most common sources of human exposure to nitroso-compounds is their formation in the acidic environment of the stomach by the reaction between electron-rich molecules present in the lumen and sodium nitrite ingested in the diet. To date, the formation of nitroso-compounds by the reaction of nitrite with food components has been investigated in depth, but little attention has been paid to substances secreted in the stomach, such as dopamine or serotonin, whose reaction products with nitrite have proven mutagenic properties. In this article, we present a kinetic study with UV–visible spectroscopy of the nitrosation reactions of both molecules, as well as of L-tyrosine, the amino-acid precursor of dopamine. We determined the kinetic parameters and reaction mechanisms for the reactions, studying the influence of the reactants concentration, pH, temperature, and ionic strength on the reaction rate. In all cases, the favoured reaction product was a stable nitroso-compound. Serotonin, the molecule whose product was the most mutagenic, underwent two consecutive nitrosation reactions. These findings suggest that additional biological research is needed to understand how this reaction alters the function of these neurotransmitters as well as the potentially toxic effects they may have once nitrosated.

## Introduction

**Figure 1:**
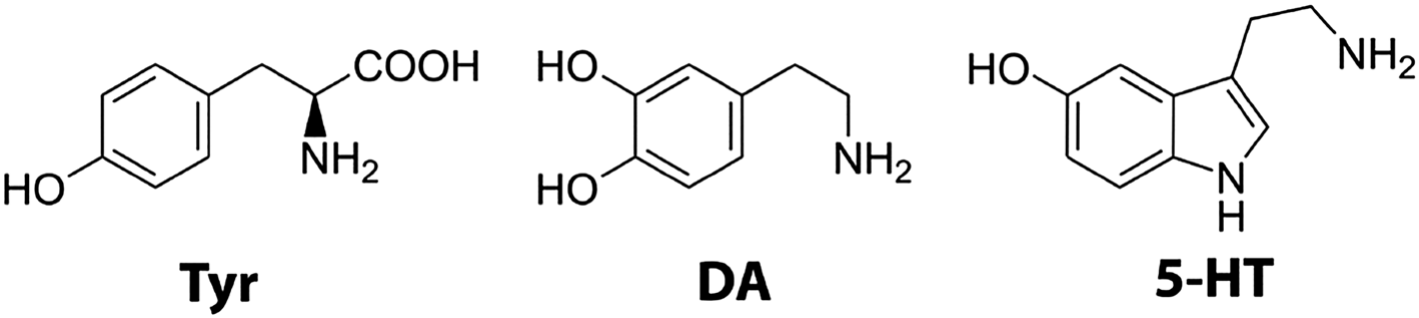
Substrates studied in this paper.

Dopamine (DA) and Serotonin (5-HT) are two neurotransmitters released into the gastric juice at the time of gastric acid secretion by the parietal and enterochromaffin cells of the stomach, respectively (Fig. 1)^1, 2^. They behave as signalling molecules that participate in the regulation of the secretions and motility of the gastrointestinal tract as the receptors^3, 4^, and although their basal concentrations in the gastric juice could be small (it was found that human stomach gastric juice contains basal concentrations of dopamine and serotonin of 4–8 ng/ml^5^ and 1–5 µg/ml^6^, respectively), they can increase considerably after food stimulation^5, 7, 8^. However, both molecules are nucleophilic, which made them susceptible to react with the derived products of sodium nitrite in the stomach to form dangerous nitroso-compounds.

Sodium nitrite (NaNO_2_) is a common preservative added to cured meat products^9^ due to its ability to enhance flavour, prevent browning due to oxidised myoglobin and protect against growth and toxin formation by *C. botulinum*^10^. But there are other sources of exposure to sodium nitrite in food^11^, like dairy products, especially cheeses, which naturally contain nitrite, or the consumption of food rich in nitrates, like vegetables (and cheeses again), that are stored in the salivary glands and saliva, where the bacteria in the oral cavity reduce them to nitrite^12^.

When nitrite is ingested and reaches the stomach, the acidic environment of the lumen transforms it partially into nitrous acid (HNO_2_), an unstable compound that rapidly turns into electrophilic species, like nitrosonium ion (NO^+^) or dinitrogen trioxide (N_2_O_3_), that react with substrates present in the stomach to form nitroso-compounds^13–15^, Nitroso-compounds are stable enough to be distributed throughout the body and to alkylate the bases of DNA, hence the studies that have been carried out on their role as carcinogen agents^16^.

Previously, it has been reported that the nitroso-compounds obtained from the reaction under acidic conditions between sodium nitrite and both dopamine and serotonin are mutagenic by means of the Ames test^17, 18^. This biological assay determines the mutagenic potential of chemical compounds by analysing their ability to induce mutations in strains of *Salmonella typhimurium* bacteria, which are measured by counting the number of colonies growing in the medium. With nitrosated dopamine, the number of colonies was 5 times higher than in the negative test suggesting that nitrosated dopamine was mutagenic^17^. With nitrosated serotonin, the number of colonies was 11 times higher than in the negative test, being nitrosated serotonin the most powerful mutagen of a large group of tryptophan metabolites studied under the same conditions^18^. Yet, the reaction mechanisms, rates and products are not known. Here, we close this gap by conducting a comprehensive kinetic investigation of the reactions of dopamine and serotonin with sodium nitrite in acidic conditions. We also present the results of the kinetic study on the nitrosation reaction of L-tyrosine (Tyr, Fig. 1), the amino acid precursor of dopamine, which surprisingly has not been done previously. This compound is released into the stomach by pepsin and other proteolytic enzymes during the digestion of food^19^, and as other amino acids, can act as a precursor of efficient alkylating agents^20^.

## Materials and methods

Serotonin hydrochloride (98%) and L-Tyrosine (99%) were purchased from Alfa Aesar. Dopamine hydrochloride (> 98%) was obtained from Sigma and deuterium oxide (99.8%) from Acros (Geel, Belgium). Sodium nitrite (ultrapure) and perchloric acid (AS) were obtained from Panreac. Sodium perchlorate (AS) was from Merck. The purity of these reagents was sufficient for the purpose of the kinetic study, eliminating the need for prior purification.

Hanna Instruments pH-211 pHmeter was used to perform pH measurements (± 0.01). Water was deionized with a Millipore MilliQGradient device.

Nitrosation reactions were monitored by determining with UV spectroscopy the amount of product generated over time. A UV–visible spectrometer (Shimadzu UV2401 PC) with a six-cell holder whose temperature was controlled by a Peltier cell (± 0.1 °C) was used for this purpose. At the lowest pHs, the reaction of nitrosation of serotonin was too fast to be followed with a traditional spectrometer. To solve this problem, we used a UV–visible stopped-flow spectrometer (Biologic SFM300) to follow it. Temperatures in this case were kept constant at 25.00 ± 0.05 °C by keeping them in a bath of water from a heating–cooling thermostat.

The initial rate method has been used for the analysis of the kinetic data of the nitrosation reaction of tyrosine and dopamine. In the case of serotonin, its kinetics has been determined using the isolation method in order to determine the characteristics of its consecutive reactions. L-tyrosine has been used without any further purification. Dopamine hydrochloride and serotonin hydrochloride were used, after checking that the chloride ion did not affect the reaction rate via nitrosyl chloride (Fig. S1). In the case of dopamine, this molecule undergoes oxidative self-polymerization under basic conditions^21^. We found that only at pH > 7.5 the rate of auto-oxidation can interfere with our kinetic study. The pH of our acidic solutions was controlled with perchloric acid (HClO_4_) and the ionic strength with sodium perchlorate (NaClO_4_), two compounds that do not catalyse the nitrosation reactions^22^.

The products were characterized using nuclear magnetic resonance (NMR) and liquid chromatography-mass spectrometry (LC–MS). UFLC separations were performed with a Thermo-Fisher Vanquish equipped with an Agilent Poroshell 120 C18 column. The flow rate was 0.2 mL/min, starting with 0.1% formic acid in water and increasing the acetonitrile concentration progressively from 1 to 50% over 8 min. After that time, 0.1% formic acid in 99% water, 1% acetonitrile, was used. The mass spectrometer was a Thermo-Fisher Q Exactive Focus Orbitrap. ^1^H NMR spectra were recorded at room temperature using Bruker models WP200-SY and Bruker Avance NEO 400 MHz with a Prodigy CPPBBO BB-H&F z-gradient cryo-probe (400 MHz to ^1^H) spectrometers.

## Results and discussion

### Nitrosation of tyrosine and dopamine

Tyrosine and dopamine reacted following the same mechanism, and therefore we will present their results in the same section. Due to their structural features, both compounds can react with nitrite through two different mechanisms: N-nitrosation of their primary amines and C-nitrosation of their phenol-activated aromatic rings^15^. In the acidic conditions of the reaction medium, the primary nitrosamines that would be produced through N-nitrosation of the ethylamine radical are highly unstable and would rapidly dissociate through diazotization, releasing nitrogen. Under the reaction conditions employed, no observable bubbles were formed in the reaction medium, suggesting that the N-nitrosation and subsequent deamination via diazonium ion of the primary amine of tyrosine and dopamine is not the main reaction^23, 24^. The change in colour of the reaction media from transparent to yellow (shift of their absorption bands from 250–300 nm to 350–400 nm, Fig. S2) is consistent with the aromatic substitution through C-nitrosation^25^.

We followed the reaction rate of tyrosine and dopamine with sodium nitrite by measuring the evolution of the absorbance at a wavelength where the reaction product absorbed with significant intensity and did not overlap with the peaks of the reactants. In the case of tyrosine, this wavelength was 410 nm and, in the case of dopamine, we used the wavelength of the maximum intensity of the absorption band of the reaction product, which appears at 347 nm (Fig. S2).

To determine the molar absorption coefficient of the product of each reaction at these wavelengths, solutions of the substrates of different concentrations were reacted with an excess of sodium nitrite. Once the saturation plateau (i.e. the point at which all the substrate in the solution has been nitrosated) was reached in each of the solutions, the values of the absorbances (*A*) were plotted against the respective initial concentrations ([Sub]_0_). It was found that, using Lambert–Beer's law

1 $$\varepsilon =\frac{A}{[Sub{]}_{0} l},$$

and taking into account that the optical path (*l*) of our cuvettes is 1 cm, the molar absorption coefficients of nitrosotyrosine (NTyr) and nitrosodopamine (NDA) were, respectively, ε_NTYR, 410 nm_ = 1550 ± 50 M^−1^ cm^−1^ and ε_NDA, 347 nm_ = 5200 ± 100 M^−1^ cm^−1^ (Fig. S3).

The initial rate method was used to determine the partial reaction orders while maintaining constant pH and ionic strength of the reaction media. We always worked under the same pH and ionic strength conditions. For each substrate, we prepared six solutions with different concentrations of substrate and the same concentration of nitrite. In this way, by taking logarithms, we can transform the rate equation to obtain the partial reaction order from the initial concentrations and measured rates:

2 $$\ln{r}_{0} = \ln p + m\,\ln [Sub{]}_{0},$$

where *r*_*0*_ is the initial rate of the reaction, *p* is a constant that contains the concentration of nitrite and the reaction constants, and [Sub]_0_ is the concentration of the substrate. *m* is the partial order of reaction for each substrate, whose values of *m* = 1.02 ± 0.03 for tyrosine (Fig. S4) and* m* = 1.02 ± 0.04 for dopamine (Fig. S5) indicate the reaction rate is first-order in substrates.

In the same way, by changing the concentrations of nitrite while keeping the concentrations of the substrates constant, we could estimate the partial reaction order for the nitrite concentration:

3 $$\ln{r}_{0} = \ln q + n\,\ln[Nit{]}_{0},$$

where *n* is the partial reaction order for the nitrite concentration and [Nit]_0_ is the initial total concentration of nitrite ([Nit] = [HNO_2_] + [NaNO_2_]), calculated from the added sodium nitrite. From our experiments, we found that *n* = 1.01 for the reaction with tyrosine (Fig. S6) and *n* = 1.00 for the reaction with dopamine (Fig. S7). These results show that both reactions are first-order in the nitrite, which rules out N_2_O_3_ as the nitrosating agent, as the reactions where this compound takes part show are second-order in nitrite^26^.

**Figure 2:**
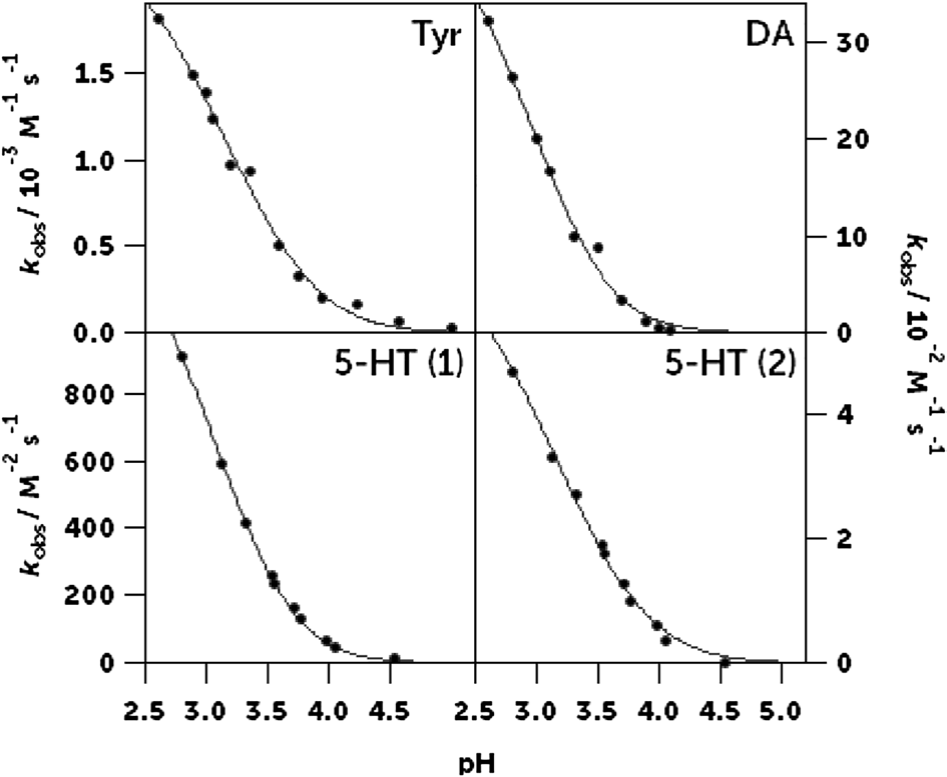
Influence of the acidity of the medium on the observed rate constant *k*_obs_. Clockwise from the top left: tyrosine, dopamine, and the second and first reactions of serotonin. The black line shows the fit of the experimental data to the rate equation derived from the proposed mechanisms. Tyrosine: [Tyr]_0_ = 7.7·10^−4^ M, [Nit] = 0.01—0.03 M, *T* = 25.0 °C, *I* = 0.20 M. Dopamine: [DA]_0_ = [Nit]_0_ = 6.0·10^−4^—2.02·10^−3^ M, *T* = 20.0 °C, *I* = 0.2 M. Serotonin: [5-HT]_0_ = 1.31·10^−4^ M, [Nit] = 3.02·10^–3^ M, *T* = 20.0 °C, *I* = 0.24 M.

Once the partial reaction orders were determined, we could study the influence of the pH and the ionic strength on the observed rate constant *k*_*obs*_:

4 $$r={k}_{obs}\left[Nit\right]\left[Sub\right],$$

by changing the concentrations of perchloric acid and sodium perchlorate in the reaction medium, respectively, and following the reaction rate. We found that ionic strength did not play a significant role in the nitrosation reaction of either of the substrates studied (Fig. S8). However, the acidity of the medium was a very influential factor in the reaction rate, causing it to increase significantly as proton concentration increased (Fig. 2).

**Figure 3:**
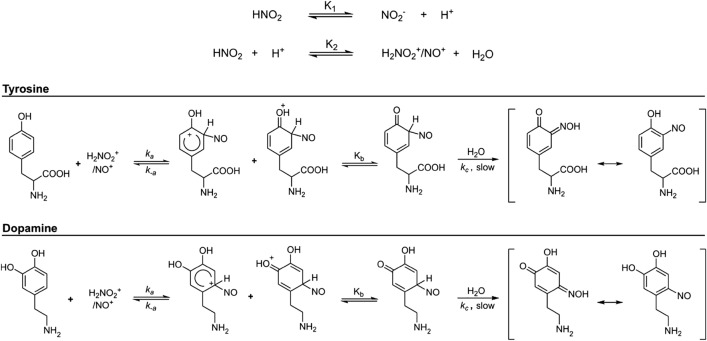
Proposed mechanism for the C-nitrosation of tyrosine and dopamine. The first step is the formation of the nitrosonium/nitrosacidium ion, which subsequently attacks tyrosine (top) or dopamine (bottom).

These results led us to propose an aromatic electrophilic substitution mechanism for the two molecules in which the effective nitrosating agents are the nitrosonium (NO^+^) or nitrosacidium (H_2_NO_2_^+^) ions, which are indistinguishable from the kinetic point of view^27^. Figure 3 shows the proposed common C-nitrosation mechanisms for tyrosine and dopamine molecules. Phenol is a strong ortho- and para-directing group in electrophilic aromatic substitutions, while the alkyl group is a weak one. In tyrosine, the para-position of the phenol is blocked, so we propose that the observed nitrosation occurs at the ortho-position of the phenol. In dopamine, there are two phenol groups. We propose that nitrosation occurs in the para-position of the phenol that has it free, because the ortho-positions of the two phenols are not favoured. This is because the ortho-positions are also the meta-positions of the other phenol. Only one aromatic substitution should occur since the nitroso group added to the aromatic ring disables any other weak aromatic substitution reaction such as an additional nitrosation reaction. Despite nitrosation in two different positions, for tyrosine and dopamine the rest of the nitrosation mechanism is the same. The rate-determining step is the deprotonation of the formed arenium ion (Wheland intermediate) that has been previously observed using transient absorption spectroscopy^28^. From the proposed mechanism, the following theoretical rate equation is obtained:

5 $$r=\frac{{K}_{2}{k}_{a}[{H}^{+}{]}^{2}[Nit][Sub]}{\left([{H}^{+}]+{K}_{1}\right)\left(1 +\frac{ {k}_{-a}}{{K}_{b}{k}_{c}} [{H}^{+}]\right)}.$$

If the proposed mechanism is correct, from the comparison of this equation with the experimental rate equation (Eq. (4)), it follows that the experimental rate constants must depend on the pH as indicated by the following equation:

6 $${k}_{obs}=\frac{\alpha [{H}^{+}{]}^{2}}{\left([{H}^{+}]+{K}_{1}\right)\left(1+\beta [{H}^{+}]\right)}$$

where *α* = *K*_*2*_* k*_*a*_ and *β* = *k*_*-a*_*/K*_*b*_* k*_*c*_. The continuous lines in Fig. 2 show the adjustment of our data to Eq. (6), having [H^+^] as a variable and using the value of *K*_*1*_ = 6.652 × 10^−4^ M obtained from the literature^29^. The good adjustment of the data and the fact that the values of *k*_*a*_ and *k*_*-a*_*/K*_*b*_* k*_*c*_ (considering that *K*_*2*_ = 3 × 10^−7^ M^−1^)^30^ are consistent with those obtained in other aromatic C-nitrosation reactions (Table 1) support our proposed mechanism.

**Table 1:** Rate and equilibrium constants and kinetic isotope effect, for the nitrosation of tyrosine, dopamine and serotonin compared with those for other C-nitrosatable compounds^32, 33^.

Substrate	*k*_*a*_ × 10^−9^ (M^−1^ s^−1^)	*k*_*-a*_*/K*_*b*_* k*_*c*_ × 10^−4^ (M^-1^)	${k}_{c}^{{H}_{2}O}/{k}_{c}^{{D}_{2}O}$	*T* / °C
Tyrosine	0.13 ± 0.04	1.7 ± 0.6	4.0	25
Dopamine	3.66 ± 0.03	0.24 ± 0.04	3.5	20
Serotonin (*k*_obs2_)	4.3 ± 0.3	1.8 ± 0.3	2.4	20
Phenol	2.2 ± 0.02	6.3 ± 0.5	3.5	25
m-cresol	2.7 ± 0.3	1.3 ± 0.1	4.4	25
o-cresol	2.4 ± 0.3	1.0 ± 0.2	3.2	25
p-cresol	0.28 ± 0.04	1.9 ± 0.3	3.0	25
2,3-dimethylphenol	3.6 ± 0.2	1.1 ± 0.2	3.0	25
2,5-dimethylphenol	7.1 ± 0.5	19.0 ± 2	4.3	25
o-bromophenol	0.27 ± 0.06	1.6 ± 0.4	1.9	25
o-chlorophenol	0.33 ± 0.03	2.0 ± 0.2	2.2	25
Minoxidil	15 ± 1	0.17 ± 0.01	8.4	25

As the rate-determining step in our proposed mechanism is the proton transfer in the Wheland intermediate, the substitution of hydrogen by deuterium must reduce the rate constant for that step (kinetic isotopic effect, KIE). By measuring the rate constant of the reaction in water and deuterated water under the appropriate pH conditions (pH = 2.1), it is possible to determine the kinetic isotopic effect associated with the constant *k*_c_. From Eq. (5):

7 $$\frac{{k}_{obs}^{{H}_{2}O}}{{k}_{obs}^{{D}_{2}O}} = \frac{{K}_{2}^{{H}_{2}O}}{{K}_{2}^{{D}_{2}O}} \frac{{k}_{c}^{{H}_{2}O}}{{k}_{c}^{{D}_{2}O}}$$

Knowing that ${K}_{2}^{{D}_{2}O}/{K}_{2}^{{H}_{2}O}$ = 2.7^31^, we determined that tyrosine and dopamine suffer a KIE of 4.0 and 3.5, respectively (Table 1).

**Figure 4:**
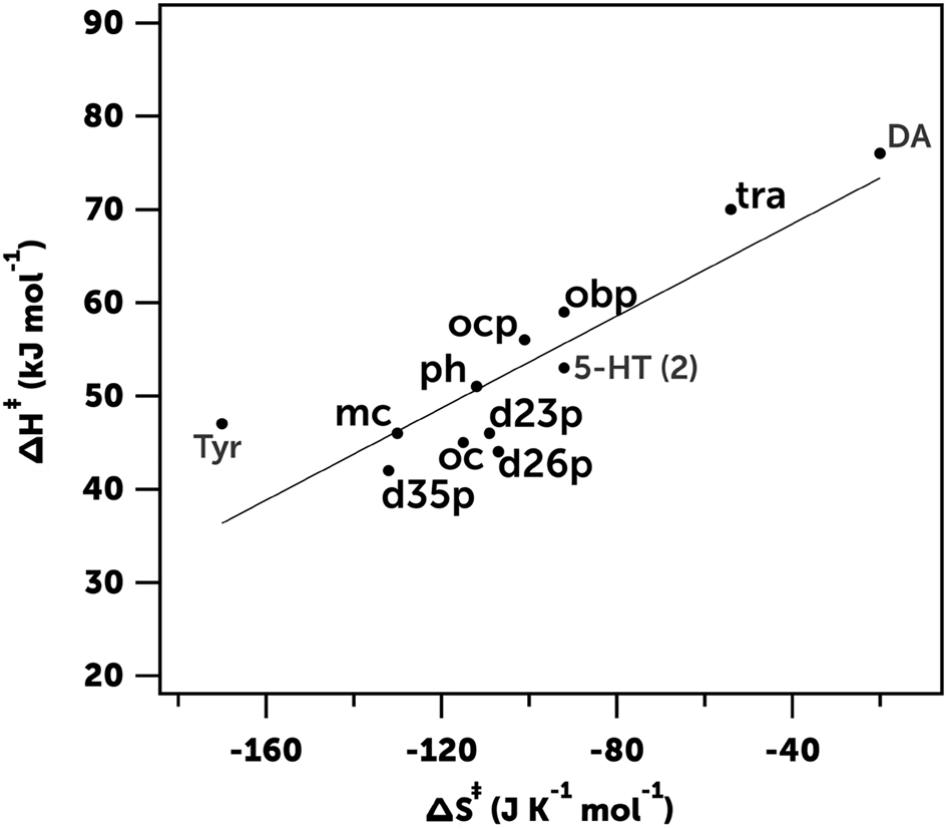
ΔH^‡^/ΔS^‡^ isokinetic relationship for the C-nitrosation reactions of tyrosine (Tyr), dopamine (DA), serotonin (5-HT (2)) and other nitrosatable substrates: phenol (ph), m-cresol (mc), o-cresol (oc), 2,3-dimethylphenol (d23p), 2,6-dimethylphenol (d26p), 3,5-dimethylphenol (d35p), o-chlorophenol (ocp), o-bromophenol (obp) and tyramine (tra)^32, 33, 36^.

To verify that these two compounds share the same mechanism among themselves and with other compounds that suffer C-nitrosation, we determined the enthalpy Δ*H*^‡^ and entropy Δ*S*^‡^ of activation of the nitrosation reactions of tyrosine and dopamine (Fig. S9) by measuring the reaction rate at different temperatures and using the equation of Eyring-Wynne-Jones^34^:

8 $$\ln\frac{{k}_{obs}}{T}=\ln\frac{{k}_{B}}{h}+\frac{\Delta {S}^{\ddagger }}{R}-\frac{\Delta {H}^{\ddagger }}{RT}$$

where *k*_*B*_,* h*, and *R* are Boltzmann, Planck and molar gas constants, respectively. Figure 4 shows that the values obtained (Δ*H*^‡^ = 47 kJ mol^−1^ and Δ*S*^‡^ =  − 170 J K^−1^ mol^−1^ for tyrosine and Δ*H*^‡^ = 76 kJ mol^−1^ and Δ*S*^‡^ = -20 J K^−1^ mol^−1^ for dopamine) support the existence of an isokinetic relationship between all substrates consistent with the sharing of the same mechanism^35^.

The nitrosation product of dopamine was determined using LC–MS (Supplementary Note 2) and ^1^H NMR (Supplementary Note 3). A reaction mixture of dopamine (8.0 × 10^–4^ M), sodium nitrite (8.0 × 10^–4^ M), and perchloric acid (5.5 × 10^–4^ M) in water was prepared. After 270 min of reaction, the sample was analysed by LC–MS. A peak at t = 4.5 min with a mass-to-charge ratio (m/z) of 199.07 was observed, corresponding to oxidized nitrosodopamine. The next day, the remaining sample was lyophilized, but not completely dried to avoid the formation of organic perchlorate salts, which are explosive. The lyophilizate was dissolved in D_2_O and the ^1^H NMR spectrum was measured after removing most of the water signal with a water suppression method. As result we obtained a spectrum where two protons appeared in the ortho- positions of the phenols (singlets at 6.6 and 7.4 ppm), along with corresponding triplets from two side-chain CH_2_ groups at 2.9 and 3.0 ppm. This is consistent with a nitrosated dopamine at the para- position, as shown in Fig. 3.

### Nitrosation of serotonin

**Figure 5:**
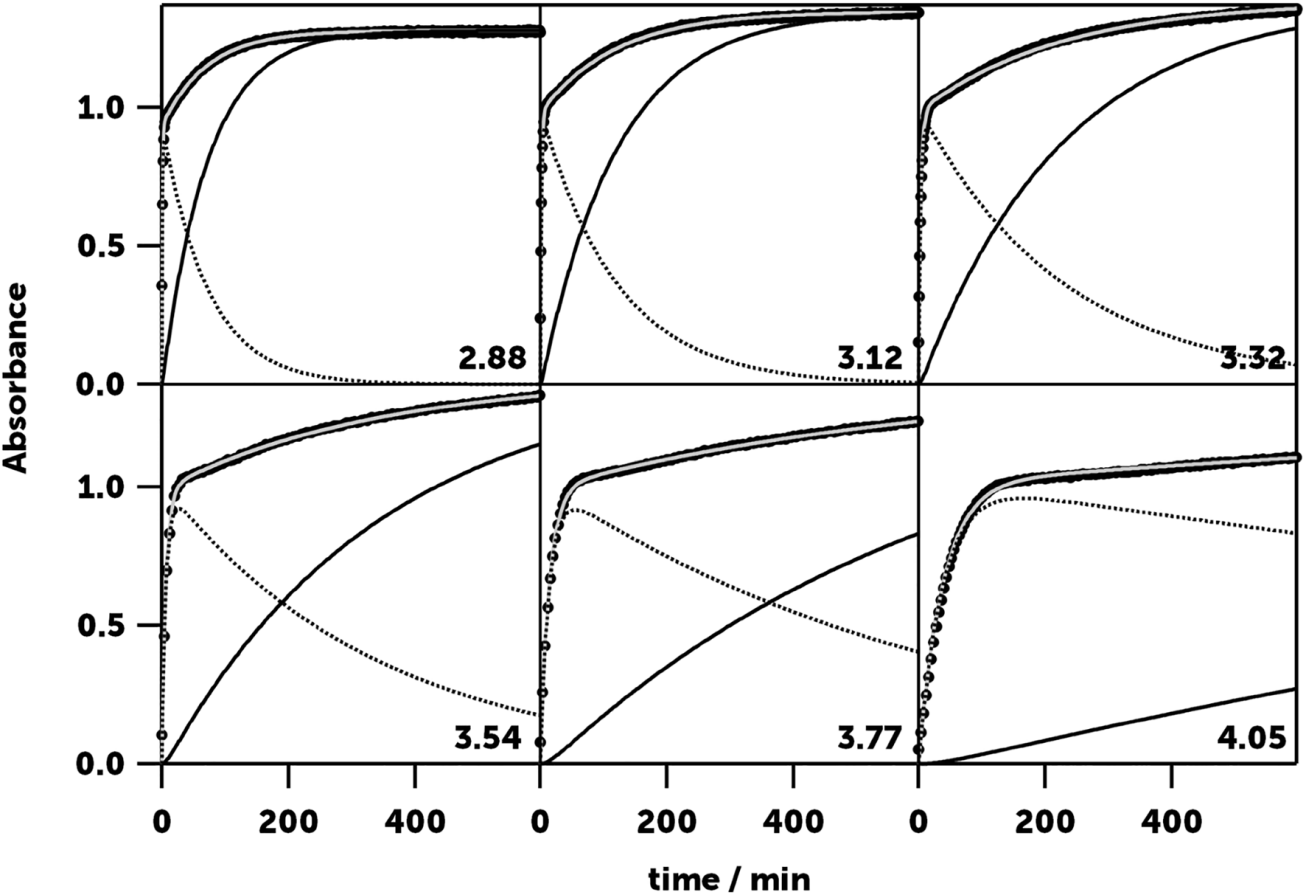
Kinetic profile of the nitrosation reactions of serotonin at different pHs and their fit to Eq. (9). The total absorbance, *A*_371_, is shown with a grey line, the absorbance of nitrososerotonin (A_B_) with a dashed black line, and the absorbance of dinitrososerotonin (A_C_) with a solid black line. [5-HT]_0_ = 1.31·10^−4^ M, [Nit] = 3.02·10^−3^ M, *T* = 20.0 °C.

Like tyrosine and dopamine, serotonin reacts under acidic conditions with nitrite showing a colour change in the reaction mixture (Fig. S2) and absence of nitrogen bubbles, so we could rule out N-nitrosation of the primary amine in the aminoethyl chain of serotonin. However, following the reaction kinetics through the absorbance of the maximum of the product band (λ = 371 nm), we found that, unlike tyrosine and dopamine, serotonin reacts through two consecutive nitrosation reactions, because despite the rapid formation of the product, a plateau in the expected saturation curve is not reached, but the absorbance continues to increase for hours (Fig. 5). The nitrous group is a strong deactivating group for aromatic electrophilic substitutions and in aromatic monocyclic compounds, given the low effectiveness of nitrite as an electrophilic agent, a second nitrosation is not observed in mild acidity and temperature conditions, even with highly activated substrates^33^. However, the indole ring of serotonin has sufficient charge density to undergo a second nitrosation reaction under the conditions in which we conducted our experiments.

As the kinetic treatment of consecutive reactions is mathematically complex, especially in the case of this nitrosation reaction in which we did not know the partial orders of the nitrite (these reactions can be second-order or third-order if the nitrosating agent is nitrosonium ion/nitrosacidium or dinitrogen trioxide, respectively)^15^, we increased the concentration of nitrite so that it had a clear excess, to create, with the isolation method, a succession of reactions of pseudo-order one:

$$\text{5-HT}\xrightarrow{{\text{H}}^{+}\text{, Nit}, {k}_{1}}\text{5-HT-NO}\xrightarrow{{\text{H}}^{+}\text{, Nit}, {k}_{2}}\text{5-HT}{\text{-(NO)}}_{2}$$

With this solution, the mathematical treatment is simple, and we can derive an expression (Supplementary Note 1) relating how the absorbance changes over the reaction time from *k*_1_ and *k*_2_. For further clarity, we will name serotonin as A, nitrososerotonin (5-HT-NO) as B, and dinitrososerotonin (5-HT-(NO)_2_) as C:

9 $${A}_{371}={\varepsilon }_{B}l\frac{{k}_{1}[A{]}_{0}}{{k}_{2}-{k}_{1}}\left({e}^{-{k}_{1}t}-{e}^{-{k}_{2}t}\right)+{\varepsilon }_{C}l[A{]}_{0}\left(1-\frac{1}{{k}_{2}-{k}_{1}}\left({k}_{2}{e}^{-{k}_{1}t}-{k}_{1}{e}^{-{k}_{2}t}\right)\right),$$

where *A*_*371*_ is the absorbance, [A]_0_ is the initial concentration of serotonin, *l* the optical path of the cuvette, and ε_B_ and ε_C_ are the molar absorption coefficients of nitrososerotonin and dinitrososerotonin. To check that the proposed mechanism of consecutive reactions agrees with the kinetic curves measured in serotonin nitrosation, several experiments were performed at different pHs. The excellent fit of Eq. (9) to the experimental data (Fig. 5) confirmed our hypothesis.

We used this method to determine all the parameters of the nitrosation reactions. To simplify the analysis, we determined first the molar absorption coefficients of nitrososerotonin and dinitrososerotonin by preparing six experiments in which the serotonin concentration was modified, keeping constant the nitrite concentration and the rest of the parameters such as pH, ionic strength and temperature. Using Eq. (9) we determined the absorbance of each product and, with the Lambert–Beer law, we obtained that ε_B, 371 nm_ = 7500 ± 80 M^−1^ cm^−1^ and ε_C, 371 nm_ = 10,100 ± 100 M^−1^ cm^−1^ (Fig. S10).

In the same way, by measuring how *k*_1_ and *k*_2_ changed as we modified the excess nitrite concentration, we obtained the partial orders with respect to the nitrite concentration of the two reactions (Fig. S11) and determined the influence of the other kinetic parameters (pH: Fig. 2; ionic strength: Fig. S8; and temperature: Fig. S9) by changing each one while keeping the rest constant. The results for each of the two nitrosation reactions are shown below.

The first nitrosation reaction of serotonin has the following experimental rate equation:

10 $$r = k_{obs1} \,\left[ {{5} - {\text{HT}}} \right]\left[ {{\text{Nit}}} \right]^{2} .$$

**Figure 6:**
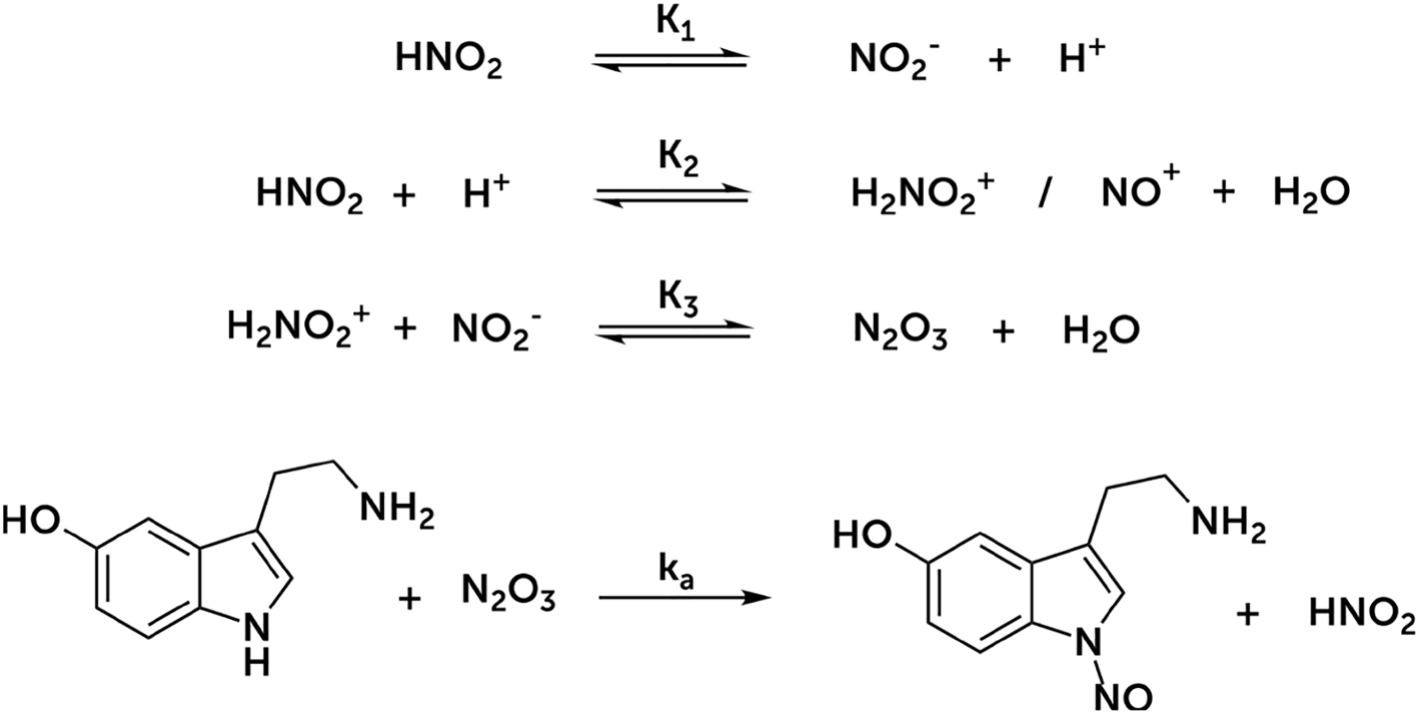
Proposed mechanism for the first nitrosation of serotonin: the N-nitrosation of the nitrogen of the indole ring of the molecule.

The observed experimental rate constant does not depend on the ionic force of the medium (Fig. S8) but is strongly influenced by the pH (Fig. 2). Order 2 with respect to nitrite concentration suggests that the nitrosating agent is dinitrogen trioxide (N_2_O_3_). Since this compound is associated with amine nitrosation^15^, we have proposed a mechanism of N-nitrosation in which the limiting stage of the rate is the N_2_O_3_ attack on the nitrogen of the indole ring (Fig. 6). The formation of a secondary nitrosamine as a result of this N-nitrosation can have further implications regarding the extraordinary mutagenic potential of this kind of compounds^14^. From this mechanism, we can deduce the following rate equation, which, unlike other N-nitrosations^20, 36, 37^, does not depend on the acidity constant of the amine, given the aromatic heteroatom condition.

11 $$r={k}_{a}{K}_{3}{K}_{2}{K}_{1}\frac{[Nit{]}^{2}[{H}^{+}][5-HT]}{\left([{H}^{+}]+{K}_{1}\right)^{2}}.$$

From the comparison of the experimental (Eq. (10)) and theoretical (Eq. (11)) rate equations, it immediately follows that:

12 $${k}_{obs1}=\alpha \frac{[{H}^{+}]}{\left([{H}^{+}]+\beta \right)^{2}},$$

where *α* = *k*_*a*_*K*_3_*K*_2_*K*_1_ and *β* = *K*_1_. The values of these parameters, according to the fitting shown in Fig. 2, are *α* = 1430 ± 30 M^−2^ s^−1^ and *β* = (40 ± 1) × 10^−5^ M. The value of the parameter *β* is useful as a probe that the fitting is chemically correct since it is the acid dissociation constant of nitric acid (p*K*_1_ = 3.3 + 0.2), which is consistent with the bibliographic value of p*K*_*a*_ = 3.177^29^. With *α*, using the Markovits constant (*K*_3_*K*_2_*K*_1_ = 3.03 × 10^−3^ M^−1^)^38^, *k*_*a*_ = (4.8 ± 1) × 10^5^ M^−1^ s^−1^ is calculated. This is a very high value, but two orders of magnitude below the limit to be considered a diffusion-controlled reaction^39^.

Comparison of *k*_1_ in water and in deuterated water at pH = 2.2 leads to the equation:

13 $$\frac{{k}_{obs1}^{{H}_{2}O}}{{k}_{obs1}^{{D}_{2}O}} = \frac{{k}_{a}^{{H}_{2}O}}{{k}_{a}^{{D}_{2}O}}\frac{{K}_{2}^{{H}_{2}O}}{{K}_{2}^{{D}_{2}O}}\frac{{K}_{1}^{{H}_{2}O}}{{K}_{1}^{{D}_{2}O}},$$

with ${K}_{1}^{{H}_{2}O}/{K}_{1}^{{D}_{2}O}$ = 0.647 and ${K}_{2}^{{H}_{2}O}/{K}_{2}^{{D}_{2}O}$ is found to be ${k}_{a}^{{H}_{2}O}/{k}_{a}^{{D}_{2}O}$ = 1.42^40, 41^. This value, small to correspond to the deprotonation of a Wheland complex, is close to that which, in diffusion-controlled processes, corresponds to the ratio of viscosities between water and heavy water, which is 1.25 to 25 °C.

Finally, we determined the activation enthalpy Δ*H*^‡^_a_ associated with the constant *k*_*a*_. In order to obtain this value, we have measured the activation enthalpy of the reaction ΔH^‡^ using Eq. (8) and then subtracted the enthalpy of formation of nitrogen trioxide (Δ*H*_M_ = 6.8 ± 0.6 kJ mol^−1^). The value obtained, Δ*H*^‡^_a_ = 26 ± 2 kJ mol^−1^, is close to the limit of the diffusion-controlled reactions (20 kJ mol^−1^)^42^.

Regarding the second nitrosation reaction, the observed rate equation

14 $$r={k}_{obs2}[5-HT-NO][Nit]$$

as well as the independence of the reaction rate of the ionic force (Fig. S8) and the shape of the *k*_obs2_-pH curve (Fig. 2) suggest that the second nitrosation is an electrophilic aromatic substitution in which, as with tyrosine and dopamine, the nitrosonium/nitrosacidium ion is the C-nitrosating agent, and the rupture of the Wheland intermediate is the rate-determining step.

To support our hypothesis, we verified that our experimental data on the influence of pH on reaction rate can be fitted perfectly with Eq. (6), that the *k*_a_ value obtained is consistent with other reactions of C-nitrosation (Table 1) and that when the reaction is repeated in heavy water and pH = 2.2 a KIE = 2.35 is measured, also consistent with other C-nitrosations (Table 1).

Similarly, the values of Δ*H*^‡^ = 53 ± 2 kJ mol^−1^ and Δ*S*^‡^ = − 92 ± 5 J K^−1^ mol^−1^ obtained for the second nitrosation reaction of serotonin (Fig. S9), show an isokinetic relationship with tyrosine, dopamine and the rest of compounds shown in Fig. 4, all of them substrates that react under the same mechanism.

The characterization of the reaction product of serotonin with sodium nitrite agrees with the mechanisms proposed here. A reaction mixture of serotonin (4.5 × 10^−4^ M), sodium nitrite (3.0 × 10^−3^ M), and perchloric acid (2.6 × 10^−3^ M) in water was prepared. After 270 min of reaction, the sample was analysed by LC–MS (Supplementary Note 2). Peaks at t = 5.8 min (*m/z* = 222.08) and t = 6.5 min (*m/z* = 267.07) were observed, corresponding to oxidized nitrososerotonin and dinitrososerotonin, respectively. After two days of reaction, the remaining sample was partially lyophilized. The freeze-dried sample was dissolved in D_2_O and the ^1^H NMR spectrum was measured after removing most of the water signal with a water suppression method (Supplementary Note 3). Several compounds appear in the spectrum, the most dominant being one that could correspond to dinitrososerotonin. The disappearance of the proton below 7.00 ppm in the predicted spectrum of serotonin suggests that C-nitrosation occurs in the ortho position (carbon 6). This result is compatible with the first nitrosation reaction occurring at the nitrogen of the indole ring of the molecule.

### Comparison of C- and N-nitrosation rates

Since both dopamine and serotonin have the ethylamine radical in their structure, both molecules can undergo simultaneous nitrosation of their aromatic groups as well as the amine of their ethylamine radical. As noted above, nitrosation of aromatic groups creates stable nitroso-compounds that are potentially dangerous because of their ability to alkylate DNA bases. In contrast, the nitroso-compound resulting from nitrosation of the primary amine of their structures decomposes rapidly via deamination. In order to estimate how many times faster the aromatic nitrosation reaction is, we calculated the z-ratio, which is the logarithm of the quotient between the rate equations we have determined in the present work and the rate equation of the N-nitrosation of phenethylamine^36^, an aromatic compound which possesses the same radical but does not undergo C-nitrosation. For this comparison, we have to assume that the nitrosation rates of the primary amines of dopamine and serotonin are similar to those of phenethylamine.

15 $$z={\log}_{10}\frac{{r}_{Subs}}{{r}_{Phe}}.$$

**Figure 7:**
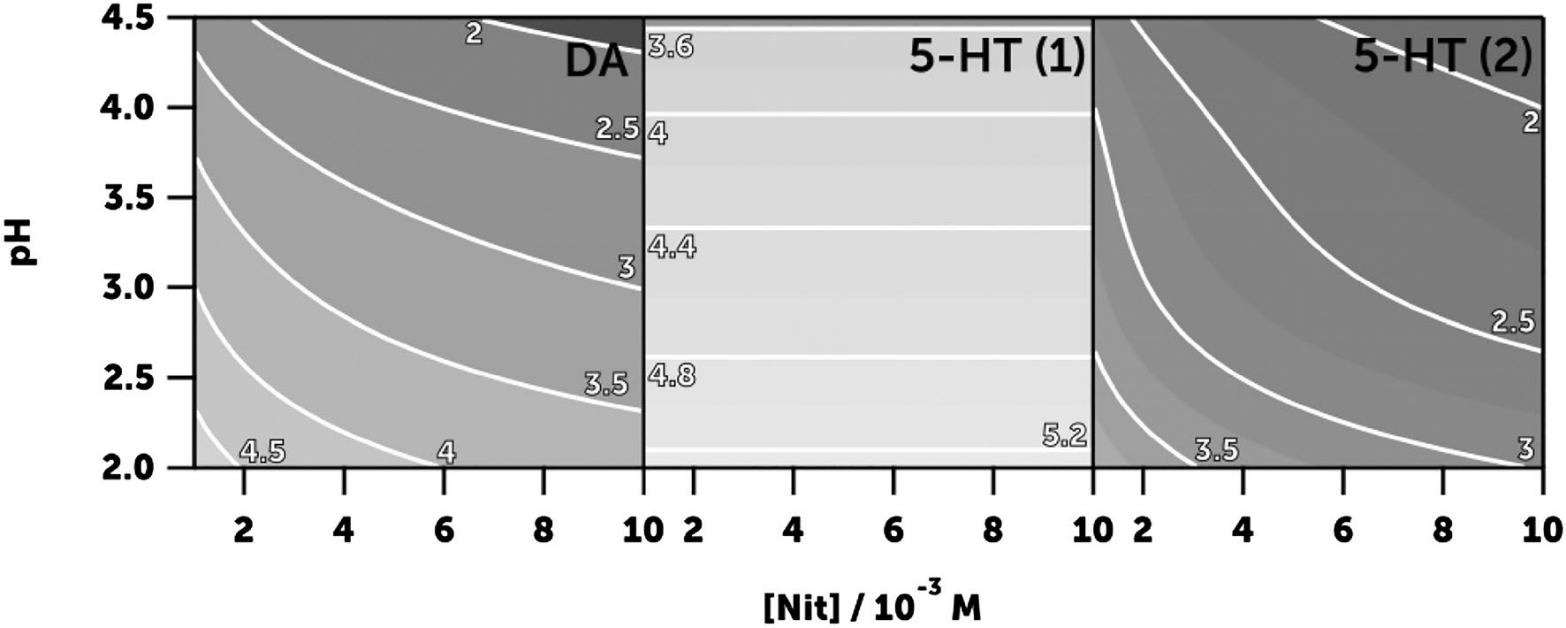
Influence of the pH and nitrite concentration on the *r*_Subs_/*r*_Phe_ ratio (Eq. (15)) of the nitrosation reactions of dopamine (left) and serotonin (first nitrosation in the middle, second at the right). The number closer to each isoline is the *z* parameter.

As we know the influence of nitrite concentration and pH on the reaction rates, it is possible to draw a contour plot showing the z-ratio for each condition. Figure 7 shows the result, where we can see that the conditions of low nitrite concentration and high acidity, common in the lumen of the stomach, greatly favour the nitrosation reaction of the aromatic groups of dopamine and serotonin, which is the reaction with the most dangerous products.

## Conclusions

1. Our results for the nitrosation of tyrosine and dopamine show that this is an electrophilic aromatic C-nitrosation reaction on their phenyl/catechol ring. We present a mechanism that explains the results obtained in which the nitrosating agent is the nitrosonium /nitrosacidium ion and the rate-limiting step is the deprotonation of the Wheland complex. We have not observed signs of N-nitrosation on the primary amine of their structures.
2. The serotonin molecule undergoes a sequential double reaction of nitrosation. The fastest reaction is the N-nitrosation of the nitrogen in the indole ring. In this reaction, the rate-determining step, very close to the limit of what is considered a diffusion-controlled reaction, is the attack of the nitrosating agent, dinitrogen dioxide, on the aromatic nitrogen. The second reaction is a C-nitrosation of the indole ring, where the electrophile is again the nitrosonium/nitrosacidium ion and the rate-determining step is the deprotonation of the arenium ion formed after the attack. As a result, a doubly nitrosated serotonin is obtained, which additionally contains a stable secondary nitrosamine that may have further implications for its mutagenic potential.
3. The high rate at which the substrates studied (especially serotonin) are transformed into compounds of proven mutagenic action, should serve to question the convenience of nitrite consumption in the diet.

### Supplementary Information

Supplementary Information.

## Data Availability

The data that support the findings of this study are available in Gredos: Research Data Repository (Universidad de Salamanca) with the identifier: http://hdl.handle.net/10366/151517.
