# Supplementary material for: Kinetic study on the reaction of sodium nitrite with neurotransmitters secreted in the stomach
Source: Sci Rep. 2023 Sep 21;13:15713. doi: 10.1038/s41598-023-42759-x (PMC10514311; doi:10.1038/s41598-023-42759-x)
Supplement: Supplementary file 1 — Supplementary Information. [file 41598_2023_42759_MOESM1_ESM.docx]

**Supplementary Information for: Kinetic study on the reaction of sodium nitrite with neurotransmitters secreted in the stomach**

Mario González-Jiménez,^a†^* M. Pilar García-Santos,^a^ Blanca Bermejo Tesón,^a^ Ángel L. Fuentes de Arriba,^b^ Jorge Arenas Valgañón,^a^ Emilio Calle,^a^ Julio Casado^a‡^

*^a^Departamento de Química Física, Universidad de Salamanca, Plaza de los Caídos, 1-5, 37008 Salamanca, Spain*

*^b^Departamento de Química Orgánica, Universidad de Salamanca, Plaza de los Caídos, 1-5, 37008 Salamanca, Spain*

*^†^ Present address: School of Chemistry, University of Glasgow, G12 8QQ Glasgow, UK.
^‡^ This author has unfortunately passed away and was unable to provide his signature for the authorship change.
*Corresponding author. Email: magonji@usal.es*

**Supplementary Notes**

**Supplementary Note 1: Derivation of the theoretical kinetic equation**

Obtaining equation 9 from a two-reaction first-order mechanism is relatively straightforward. To calculate the kinetic parameters of these reactions, one must first know the rates of formation and disappearance of each of the compounds involved.

The rate of disappearance of serotonin, A, is governed by the rate constant *k*_1_:

$-\frac{d[A]}{dt}=k_{1}[A]$ **(S1)**

Mononitrosated serotonin, B, after forming, can nitrosate again and disappear:

$\frac{d[B]}{dt}=k_{1}[A]-k_{2}[B]$ **(S2)**

In this equation, *k*_1_[A] represents the formation of B from A and the term *k*_2_[B] represents the transformation of B into C. The rate of formation of dinitrososerotonin, C, is calculated with the equation:

$\frac{d[C]}{dt}=k_{2}[B]$ **(S3)**

**Equation S1** represents a first-order process. Rearranging the equation and integrating

$\int-\frac{d[A]}{[A]}=\int k_{1}dt$ **(S4)**

we obtain how the concentration of serotonin varies over time:

$[A]=[A]_{0}e^{-k_{1}t}$ **(S5)**

where [A]_0_ is the initial concentration of serotonin, which will be converted into the different compounds:

$[A]_{0}=[A]+[B]+[C]$ **(S6)**

To find the concentration of B, the solution of **equation S1** can be used

$\frac{d[B]}{dt}=k_{1}[A]_{0}e^{-k_{1}t}-k_{2}[B]$ **(S7)**

Rearranging the terms of this equation shows that it is a first order linear differential equation:

$\frac{d[B]}{dt}+k_{2}[B]=k_{1}[A]_{0}e^{-k_{1}t}$ **(S8)**

To solve it, one can multiply both sides of the equation by the integrating factor $e^{k_{2}t}$

$\left( \frac{d[B]}{dt}+k_{2}[B] \right)e^{k_{2}t}=k_{1}[A]_{0}e^{-k_{1}t}e^{k_{2}t}$ **(S9)**

and simplify:

$\frac{d[B]}{dt}e^{k_{2}t}+k_{2}[B]e^{k_{2}t}=k_{1}[A]_{0}e^{(k_{2}-k_{1})t}$ **(S10)**

The left-hand side of the equation is equal to:

$\frac{d[B]}{dt}e^{k_{2}t}+k_{2}[B]e^{k_{2}t}=\frac{d([B]e^{k_{2}t})}{dt}$ **(S11)**

So substituting into **equation S10**:

$\frac{d([B]e^{k_{2}t})}{dt}=k_{1}[A]_{0}e^{(k_{2}-k_{1})t}$ **(S12)**

and integrating:

$\int\frac{d([B]e^{k_{2}t})}{dt}dt=\int[A]_{0}e^{(k_{2}-k_{1})t}dt$ **(S13)**

we obtain the solution of the equation:

$[B]e^{k_{2}t}=\frac{k_{1}}{k_{2}-k_{1}}[A]_{0}e^{(k_{2}-k_{1})t}+D$ **(S14)**

Where *D* is a constant of integration. To calculate its value, after clearing the concentration of B,

$[B]=\frac{k_{1}}{k_{2}-k_{1}}[A]_{0}e^{-k_{1}t}+De^{{-k}_{2}t}$ **(S15)**

the initial concentration of B at time zero is calculated. Thus, the above equation looks like this:

$[B]_{0}=\frac{k_{1}[A{]_{0}}}{k_{2}-k_{1}}+D$**(S16)**

By subtracting D and substituting in 4.97:

$[B]=\frac{k_{1}}{k_{2}-k_{1}}[A]_{0}(e^{-k_{1}t}-e^{-k_{2}t})+[B]_{0}e^{{-k}_{2}t}$ **(S17)**

In this equation, the first term on the right-hand side represents the mononitrososerotonin in the medium after its formation through nitrosation of serotonin and subsequent disappearance by another nitrosation, while the second term describes the mononitrososerotonin that is present initially. As this quantity is zero, the above expression can be simplified:

$[B]=\frac{k_{1}}{k_{2}-k_{1}}[A]_{0}(e^{-k_{1}t}-e^{-k_{2}t})$ **(S18)**

For the second reaction, the formation of dinitrososerotonin (C) from nitrososerotonin, the equation is again that of a first-order process, so its solution is immediate:

$[C]=[A]_{0}\left( 1-\frac{1}{k_{2}-k_{1}}(k_{2}e^{-k_{1}t}-{k_{1}e}^{-k_{2}t}) \right)$ **(S19)**

Having calculated the concentrations of each of the compounds in the consecutive reactions, we proceeded to fit these equations to the experimental results. At λ = 371 nm, only compounds B and C absorb. As:

$A_{B}=\varepsilon_{B}l[B]$ **(S20)**

$A_{C}=\varepsilon_{C}l[C]$ **(S21)**

the absorbance over the reaction time can be deduced:

$A_{371}=\varepsilon_{B}l\frac{k_{1}[A]_{0}}{k_{2}-k_{1}}\left( e^{-k_{1}t}-e^{-k_{2}t} \right)+\varepsilon_{C}l[A]_{0}\left( 1-\frac{1}{k_{2}-k_{1}}\left( k_{2}e^{-k_{1}t}-k_{1}e^{-k_{2}t} \right) \right)$ **(S22)**

This is **Equation 9**.

**Supplementary note 2: LC-MS**

**Nitrosation of dopamine**

| Display Formula | S Fit | RDB | Delta [mmu] | Theo. mass | Combined Score | # Matched Iso. |
| --- | --- | --- | --- | --- | --- | --- |
| C₈H₁₁O₄N₂ | 40.35 | 4.5 | -0.4 | 199.07133 | 95.85 | 4 |
| C₇H₈N₆²³Na | 15.29 | 6.5 | 0.67 | 199.07027 | 93.87 | 3 |
| C₃H₁₂O₂N₆³⁵Cl | 5.747 | 0.5 | 0.46 | 199.07048 | 86.1 | 3 |
| C₆H₁₆ON₂³⁵Cl³²S | 0 | -0.5 | 4.3 | 199.06664 | 0 | 3 |
| C₅H₁₅O₄N₂³²S | 0 | -0.5 | -3.77 | 199.0747 | 0 | 3 |
| C₃H₁₁O₆N₄ | 0 | 0.5 | 3.63 | 199.06731 | 0 | 3 |
| C₈H₁₂N₄³⁵Cl | 0 | 4.5 | -3.56 | 199.0745 | 0 | 3 |
| C₄H₁₅ON₄³²S₂ | 0 | -0.5 | 2.76 | 199.06818 | 0 | 4 |
| C₄H₁₂N₆²³Na³²S | 0 | 1.5 | -2.7 | 199.07364 | 0 | 2 |
| C₇H₁₆O₄³⁵Cl | 0 | -0.5 | -2.23 | 199.07316 | 0 | 3 |
| C₁₃H₁₁O₂ | 0 | 8.5 | -4.42 | 199.07536 | 0 | 0 |
| C₁₁H₁₂O₂²³Na | 0 | 5.5 | -2.01 | 199.07295 | 0 | 2 |

**Nitrosation of serotonin**

| Display Formula | S Fit | RDB | Delta [ppm] | Theo. mass | Combined Score | # Matched Iso. |
| --- | --- | --- | --- | --- | --- | --- |
| C₁₀H₁₂O₃N₃ | 27.18 | 6.5 | -2.09 | 222.08732 | 92.18 | 4 |
| C₅H₁₃ON₇³⁵Cl | 6.942 | 2.5 | 1.76 | 222.08646 | 81.99 | 3 |

| Display Formula | S Fit | RDB | Delta [ppm] | Theo. mass | Combined Score | # Matched Iso. |
| --- | --- | --- | --- | --- | --- | --- |
| C₁₀H₁₁O₅N₄ | 29.69 | 7.5 | -1.29 | 267.0724 | 92.96 | 4 |
| C₁₀H₁₉O₄³²S₂ | 26.21 | 1.5 | 0.47 | 267.07193 | 92.77 | 3 |
| C₉H₁₅O₉ | 19.37 | 2.5 | 3.72 | 267.07106 | 91.67 | 2 |
| C₉H₈ON₈²³Na | 17.76 | 9.5 | 2.71 | 267.07133 | 91.58 | 4 |
| C₁₂H₂₁⁷⁹Br²³Na | 16.56 | 1.5 | 0.63 | 267.07188 | 91.52 | 2 |
| C₁₃H₁₆N₂³⁵Cl³²S | 10.73 | 6.5 | 1.23 | 267.07172 | 91.21 | 2 |
| C₁₁H₁₅N₄³²S₂ | 10.4 | 6.5 | -4.54 | 267.07326 | 91.19 | 2 |
| C₅H₁₆O₅N₄²³Na³²S | 10.95 | -0.5 | -4.9 | 267.07336 | 83.1 | 3 |
| C₇H₁₇O₅N₂³⁵Cl²³Na | 10.89 | -0.5 | 0.87 | 267.07182 | 83.1 | 4 |
| C₅H₁₂O₃N₈³⁵Cl | 5.97 | 3.5 | 1.92 | 267.07154 | 82.09 | 4 |
| C₃H₁₁O₃N₁₀³²S | 5.153 | 3.5 | -3.85 | 267.07308 | 82.05 | 3 |
| C₉H₁₆N₄²³Na³²S₂ | 4.389 | 3.5 | 4.47 | 267.07086 | 82.01 | 3 |

**Supplementary note 3: ^1^H NMR**

**Nitrosation of dopamine**

**^1^H NMR of reaction medium after one day**

**
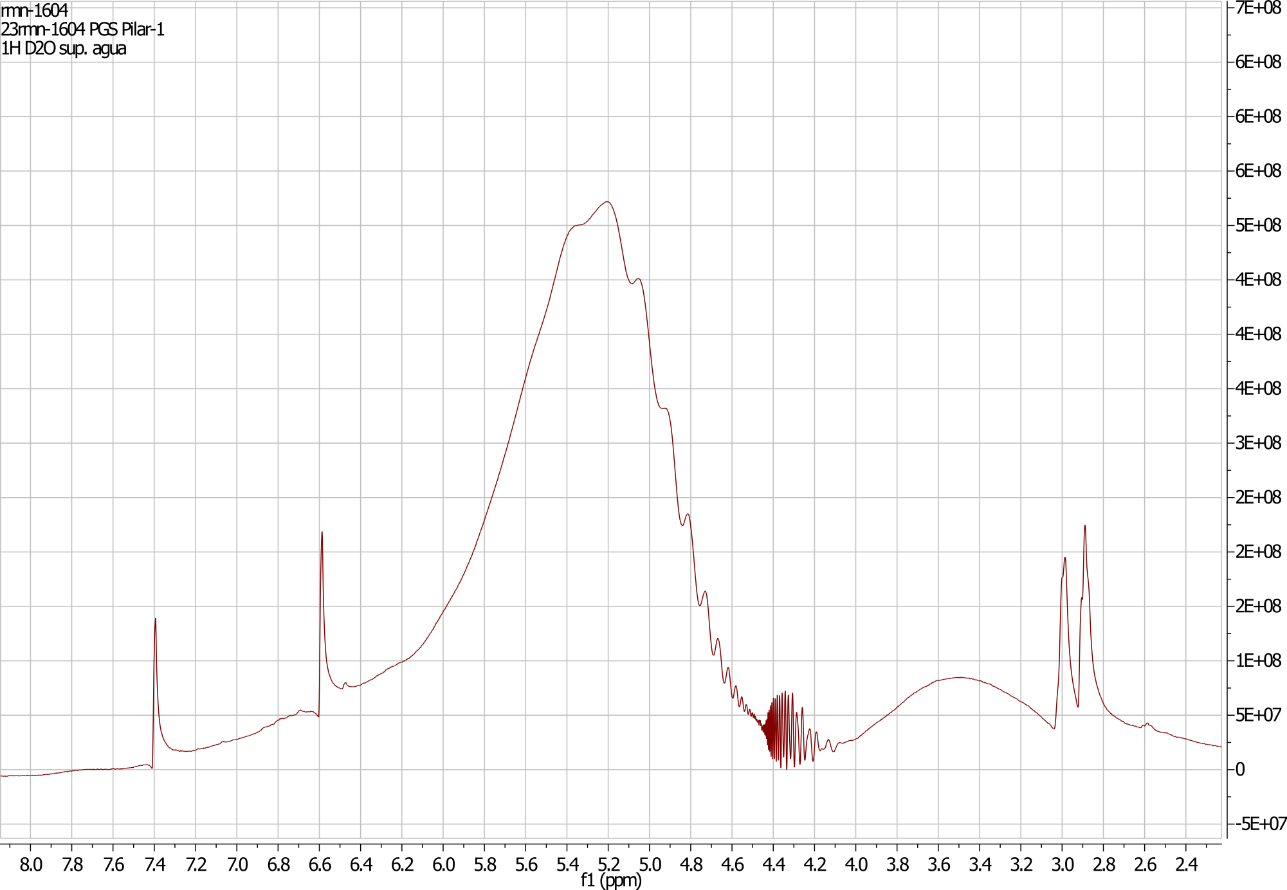
**

**Nitrosation of serotonin**

**Predicted ^1^H NMR of serotonin**

**
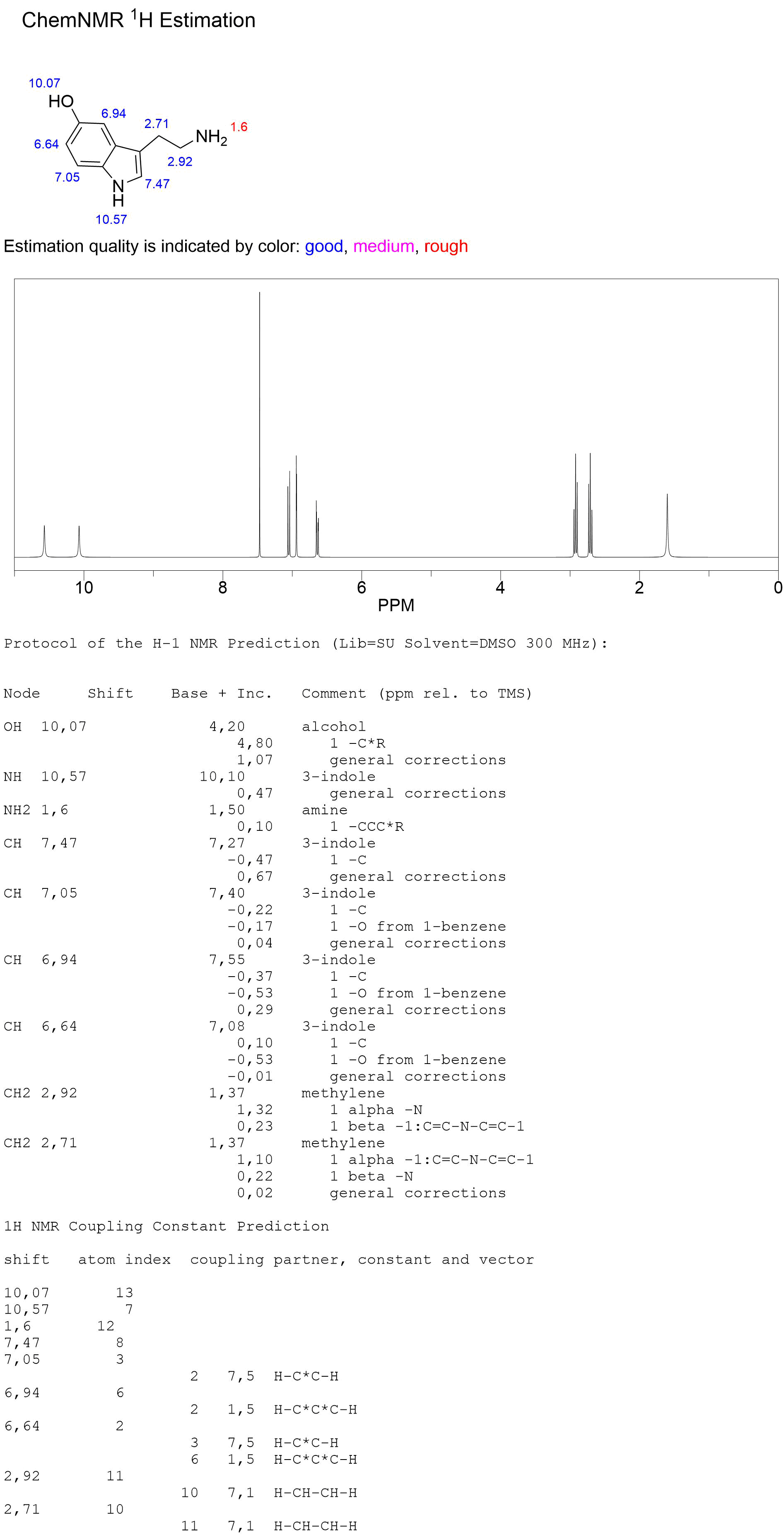
**

**Predicted ^1^H NMR of dinitrososerotonin**

**
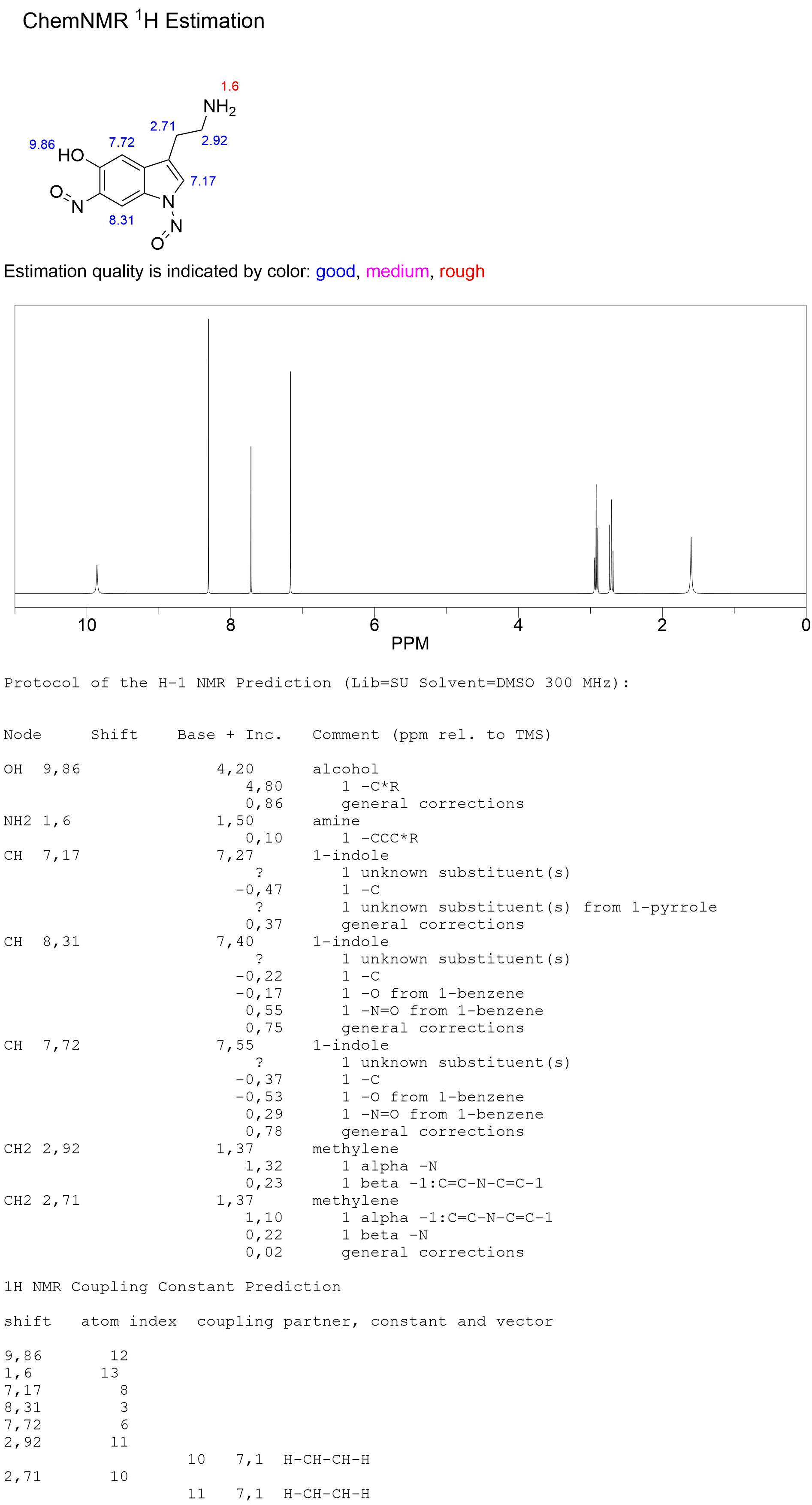
**

**^1^H NMR of reaction medium after two days**

**
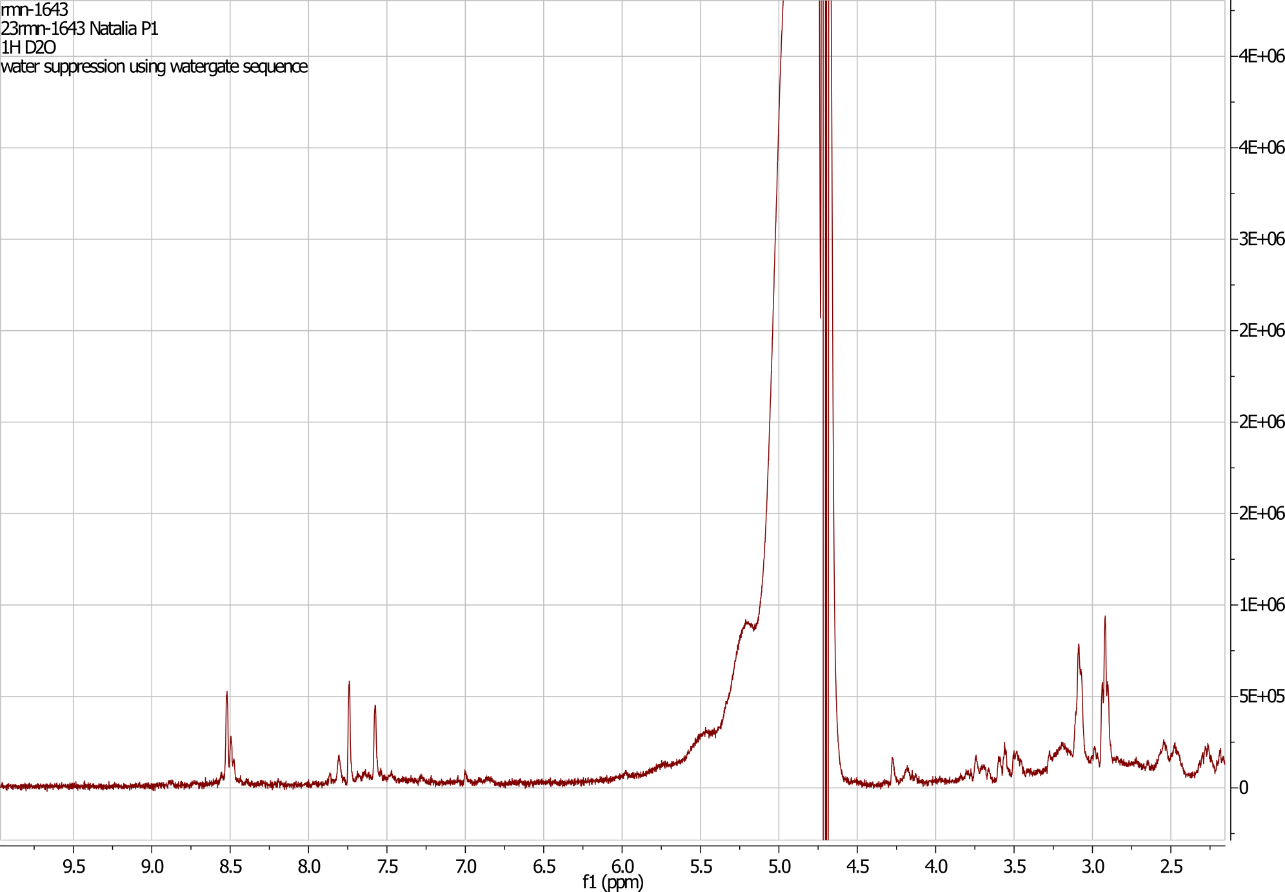
**

**Supplementary Figures**

**
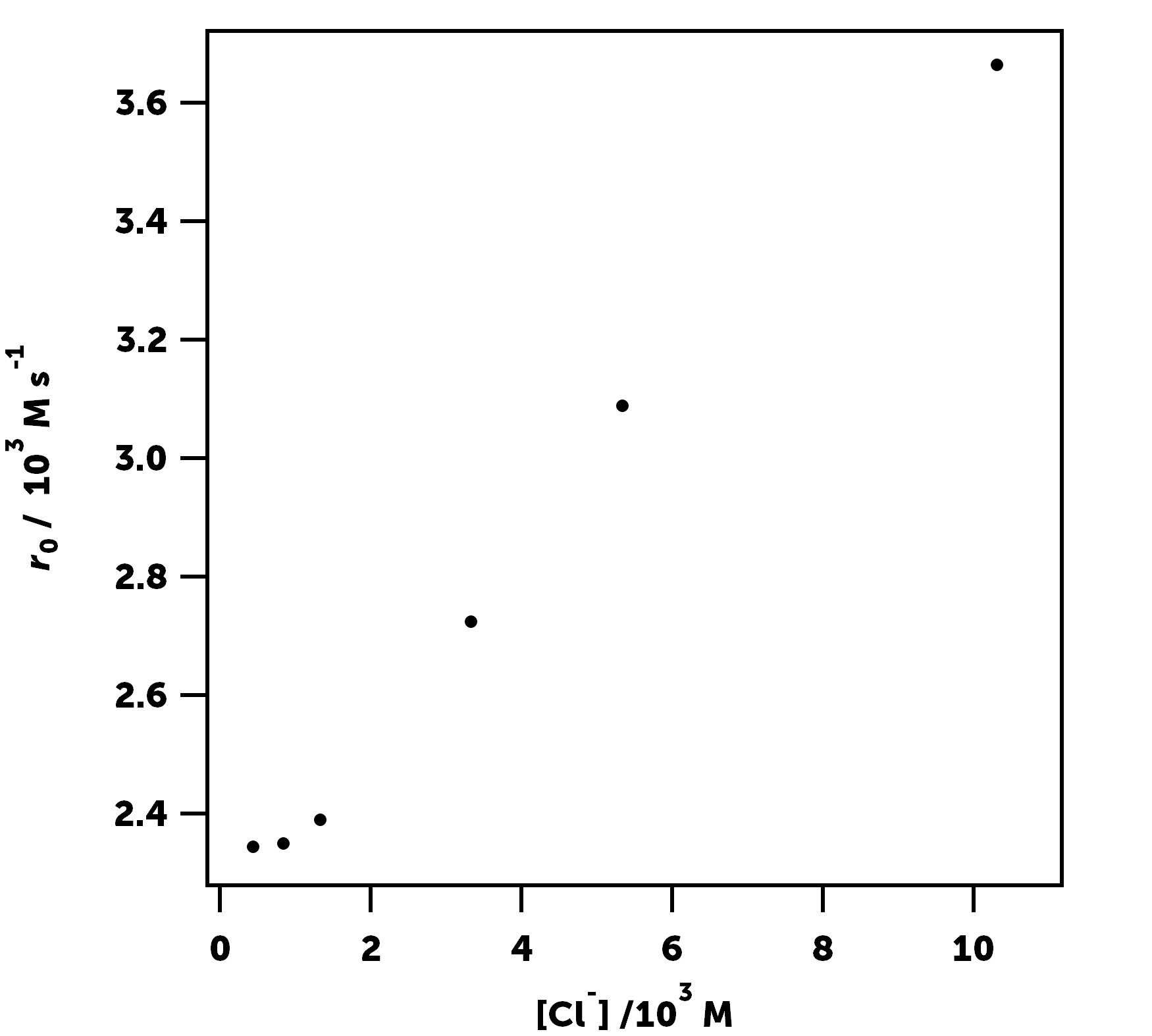
**

**Figure S1.** Influence of the concentration of chloride ions on the dopamine nitrosation initial rate. [DA]_0_ = 3.33·10^-4^ M, [Nit]_0_ = 6.03·10^-4^ M, pH = 4.08, *T* = 20.0 °C


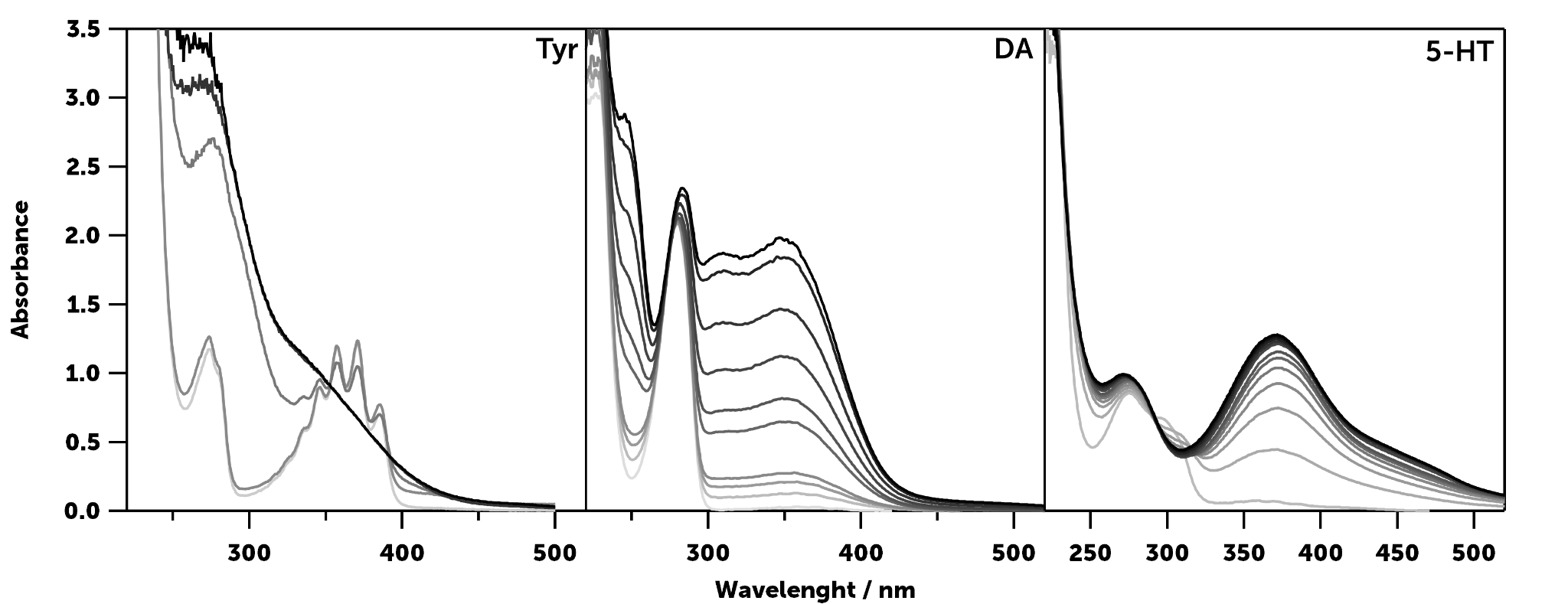


**Figure S2:** Variation over time of the absorption spectrum of tyrosine (left), dopamine (centre), and serotonin (right) during its nitrosation. The reactions were followed during 64 h, 6 h, and 5 h, respectively. The spectrum at t=0 is shown in light grey and the time progression is shown darkening the shades. [Tyr] = 7.77·10^-4^ M, [Nit] = 3,00·10^-2^ M, pH = 3.0, *T* = 26.0 °C (left). [DA] = [Nit] = 8·10^-3^ M, pH = 4.08, *T* = 25.0 °C (centre). [5-HT] = 3.03·10^-4^ M, [Nit] = 3.01·10^-3^ M, pH = 3.90, *T* = 20.0 °C (right).

**
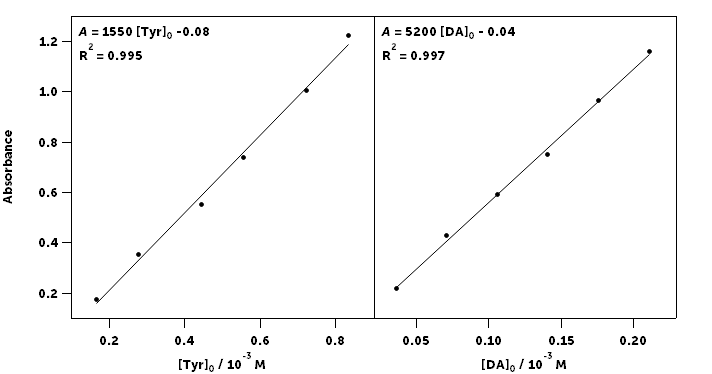
**

**Figure S3.** Determination of the absorption coefficient of the nitrosation product of tyrosine (left) and dopamine (right). Tyrosine: λ = 410 nm, [Nit] = 3.35·10^-2^ M, pH = 3.6, *T* = 25.0 °C (left). Dopamine: λ = 347 nm, [Nit] = 5.2·10^-2^ M, pH = 4.16, *T* = 25.0 °C (right).


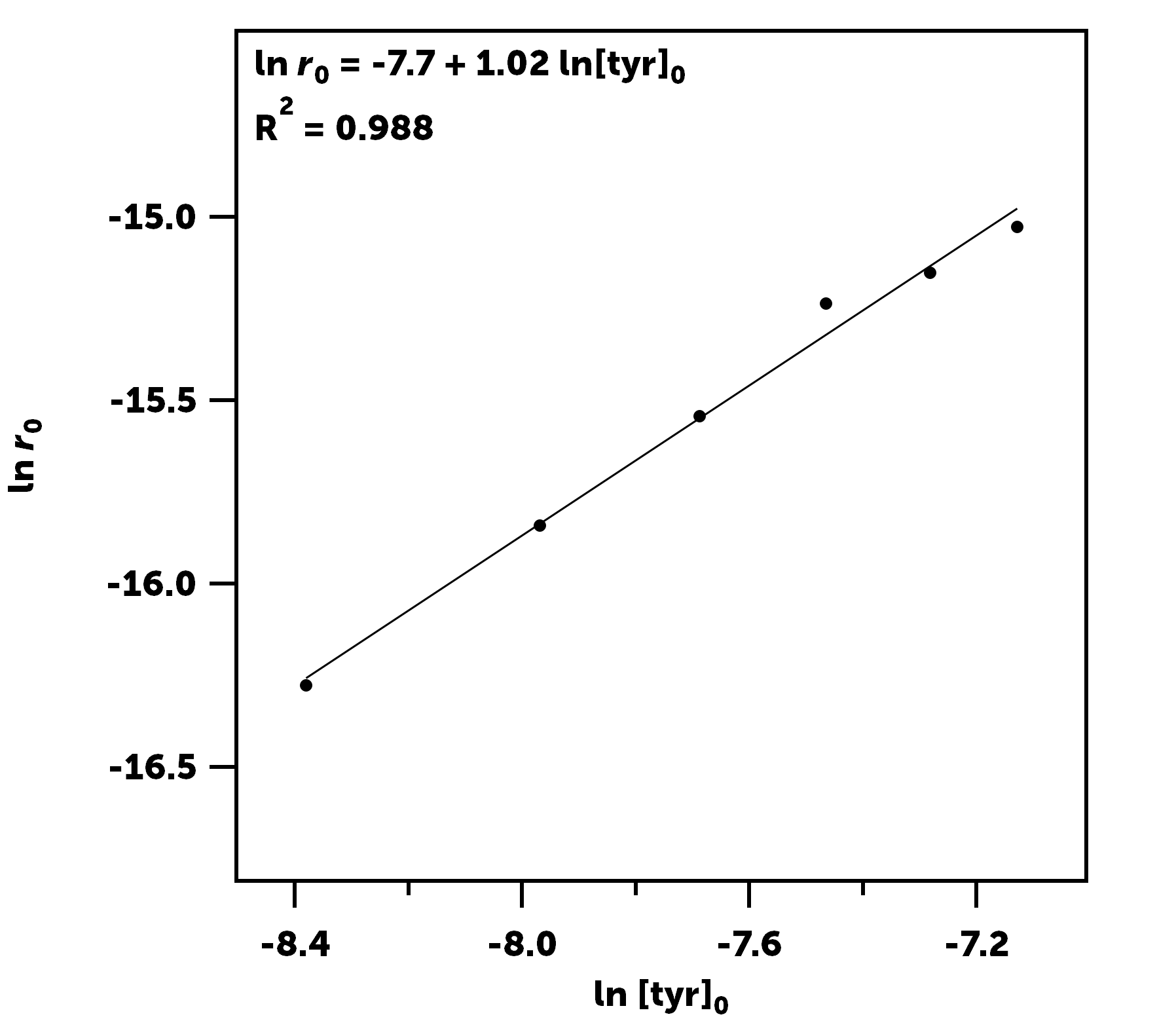


**Figure S4.** Determination of the partial reaction order for tyrosine. [Nit]_0_ = 3.05 · 10^-2^ M, pH = 3.8, *T* = 25.0 °C, *I* = 0.20 M.


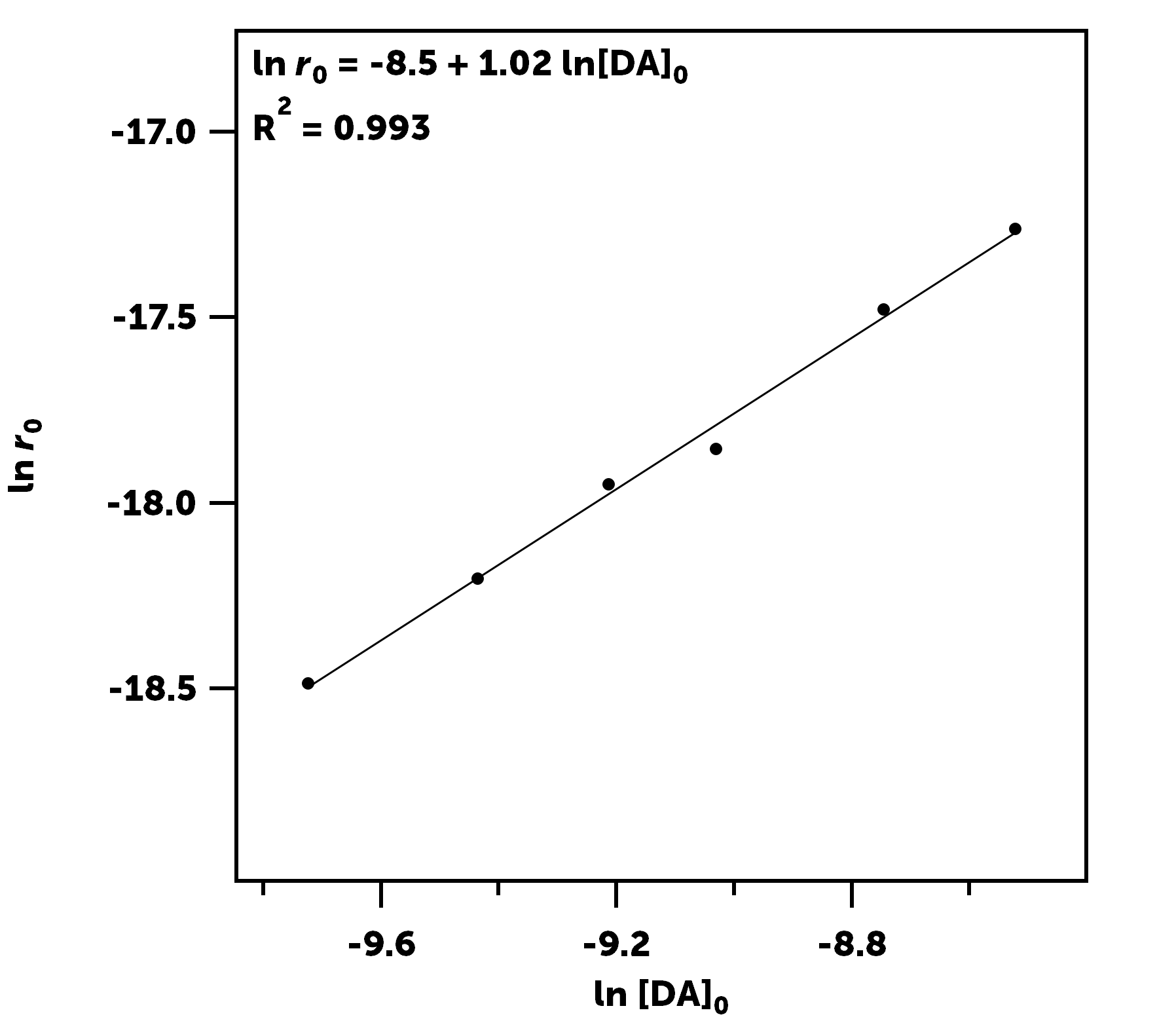


**Figure S5.** Determination of the partial reaction order for dopamine. [Nit]_0_ = 1.20 · 10^-3^ M, pH = 4.01, *T* = 20.0 °C, *I* = 0.20 M.


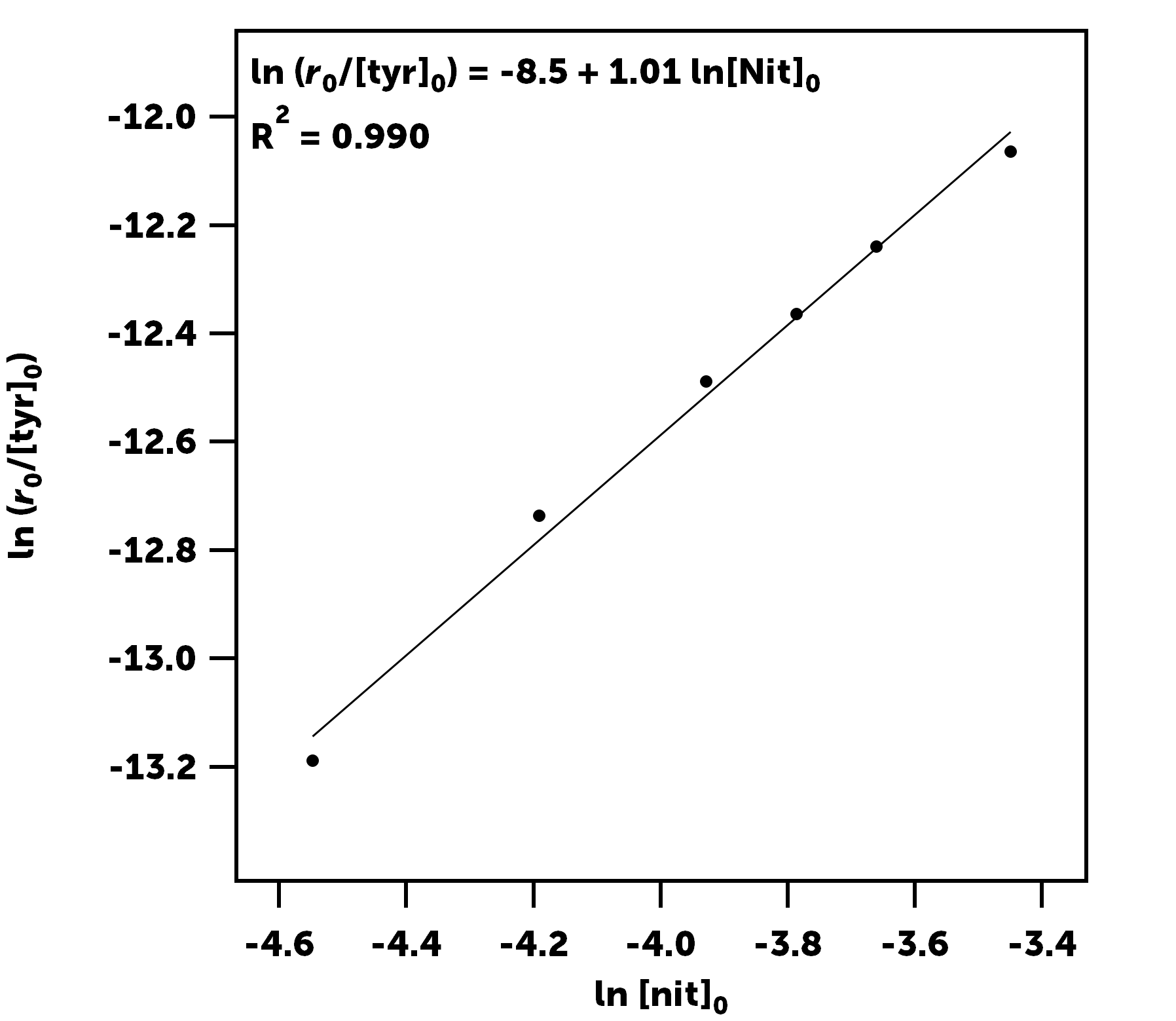


**Figure S6.** Influence of the nitrite concentration on the initial reaction rate of tyrosine nitrosation. [Tyr]_0_ = 7.76 · 10^-4^ M, pH = 3.9, *T* = 25.0 °C, *I* = 0.20 M.


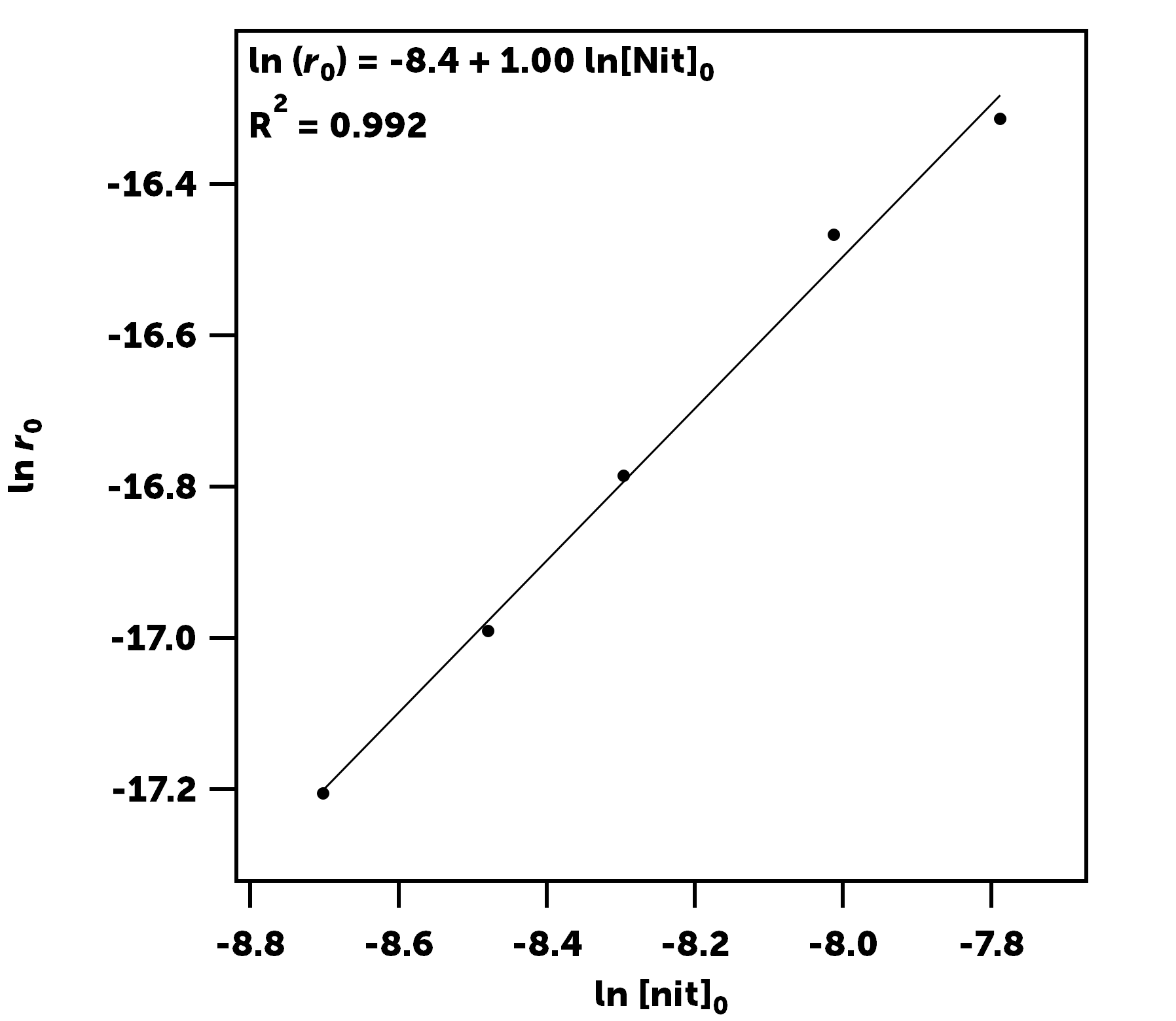


**Figure S7.** Influence of the nitrite concentration on the initial reaction rate of dopamine nitrosation. [DA]_0_ = 1.32 · 10^-3^ M, pH = 3.98, *T* = 20.0 °C, *I* = 0.20 M.


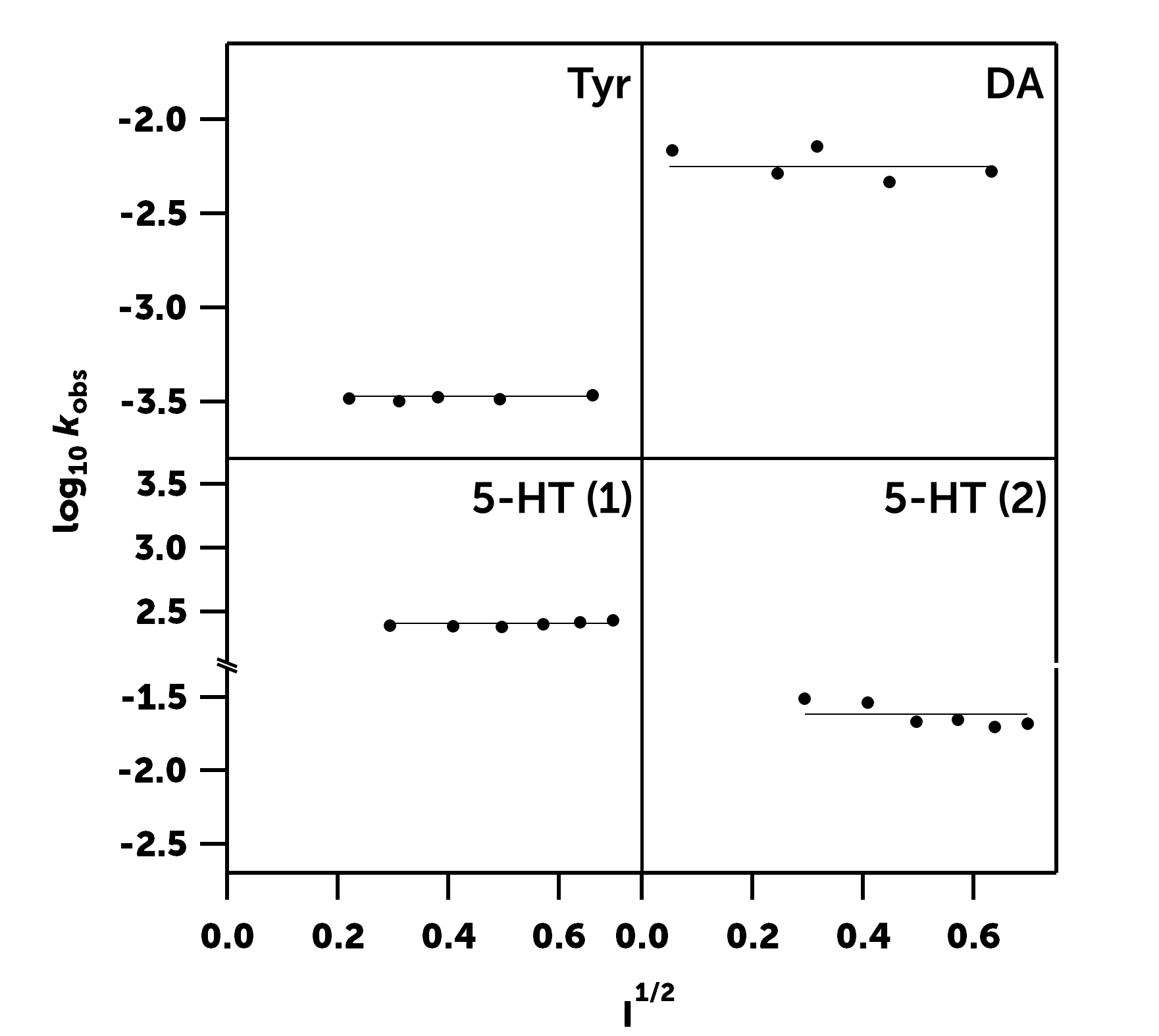


**Figure S8.** Influence of ionic strength on the observed rate constant *k*_obs_. Also shown horizontal lines to guide the eye. Clockwise from top left: tyrosine, dopamine, and the second and first reactions of serotonin. Tyrosine: [Tyr]_0_ = 7.72·10^-4^ M, [Nit]_0_ = 1.06·10^-2^ - 3.18·10^-2^ M, pH = 3.76, *T* = 25.0 °C. Dopamine: [DA]_0_ = [Nit]_0_ = 6.0·10^-4^ - 2.02·10^-3^ M, pH = 4.0, *T* = 20.0 °C. Serotonin: [5-HT]_0_ = 1.53·10^-4^ M, [Nit] = 6.03·10^-3^ M, pH = 3.50, *T* = 20.0 °C.


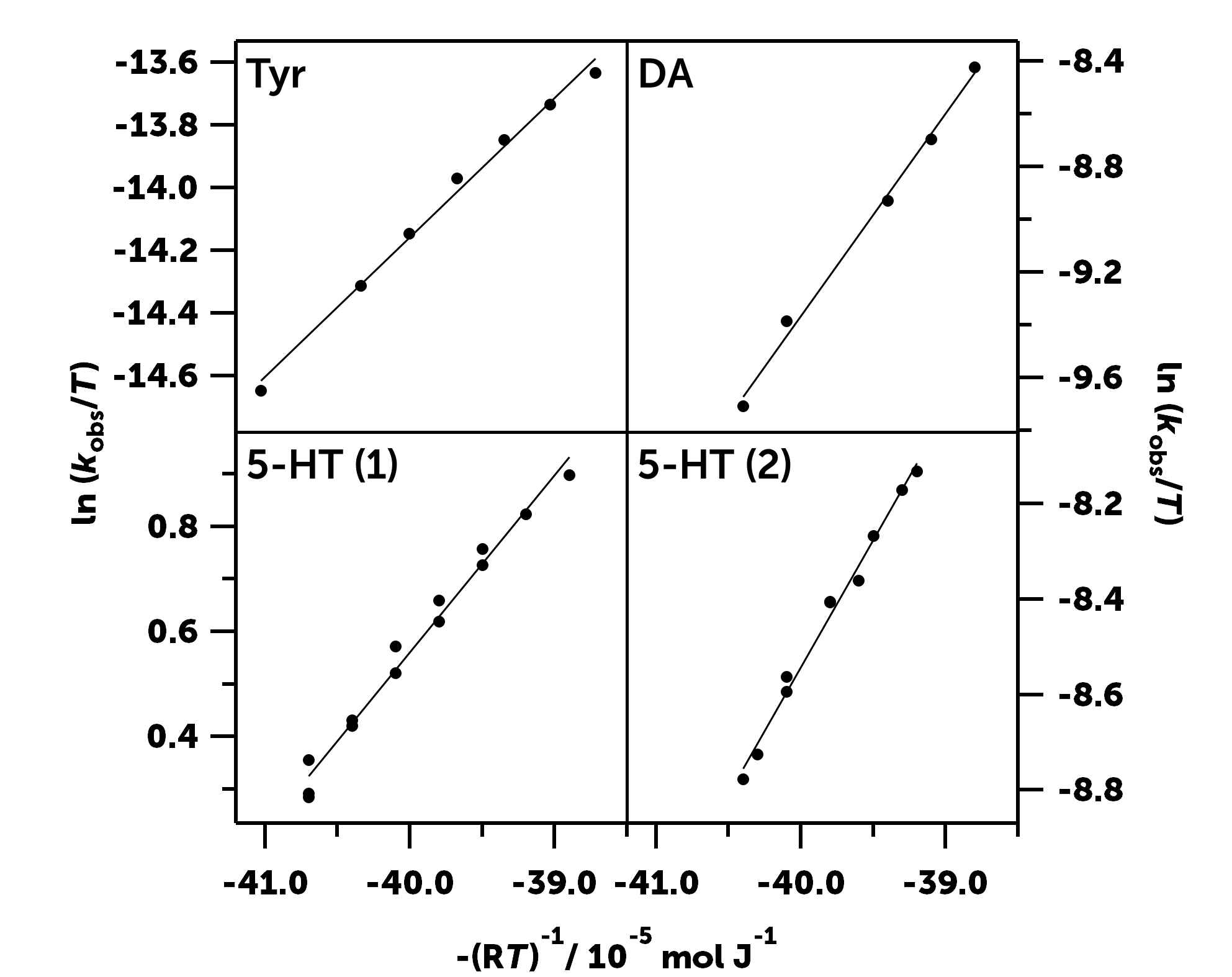


**Figure S9.** Fitting of the observed rate constant in the nitrosation reactions of tyrosine, dopamine and serotonin at different temperatures to the Eyring-Wynne-Jones equation (**Equation 8**). Tyrosine: [Tyr]_0_ = 7.7·10^-4^ M, [Nit]_0_ = 0.01-0.03 M, pH = 3.5, *I* = 0.2 M. Dopamine: [DA]_0_ = [Nit]_0_ = 6.0·10^-4^ - 2.01·10^-3^ M, pH = 3.76, *I* = 0.2 M. Serotonin: [5-HT]_0_ = 3.78·10^-5^ - 1.28·10^-4^ M, [Nit] = 3.00·10^-3^ M, pH = 3.65.


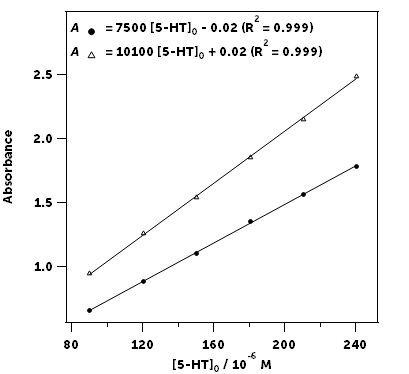


**Figure S10**. Compliance with the Lambert-Beer law by nitrososerotonin (black circles) and dinitrososerotonin (white triangles). [Nit] = 6.028·10^-3^ M, pH = 4.02, *T* = 20.0 °C.


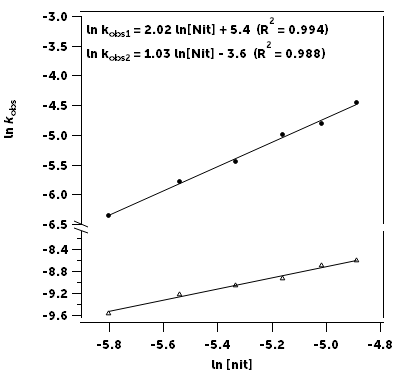


**Figure S11.** Influence of nitrite concentration on the rate constants *k*_obs1_ (black circles) and *k*_obs2_ (white triangles). [5-HT]_0_ = 1.53·10^-4^ M, pH = 4.00, *I* = 0.24M, *T* = 20.0 °C.
